# Epigenetic architecture of *Pseudotaxus chienii*: Revealing the synergistic effects of climate and soil variables

**DOI:** 10.1002/ece3.10511

**Published:** 2023-09-10

**Authors:** Yingjuan Su, Li Liu, Qi Deng, Zhuyan Lü, Zhen Wang, Ziqing He, Ting Wang

**Affiliations:** ^1^ School of Life Sciences Sun Yat‐sen University Guangzhou China; ^2^ Research Institute of Sun Yat‐sen University in Shenzhen Shenzhen China; ^3^ School of Medicine Guangxi University of Science and Technology Liuzhou China; ^4^ College of Life Sciences South China Agricultural University Guangzhou China

**Keywords:** climate and soil variables, MSAP, population epigenetic variation, *Pseudotaxus chienii*, synergistic effects

## Abstract

Whether conifers can withstand environmental changes especially temperature fluctuations has been controversial. Epigenetic analysis may provide new perspectives for solving the issue. *Pseudotaxus chienii* is an endangered gymnosperm species endemic to China. In this study, we have examined the genetic and epigenetic variations in its natural populations aiming to disentangle the synergistic effects of climate and soil on its population (epi)genetic differentiation by using amplified fragment length polymorphism (AFLP) and methylation‐sensitive AFLP (MSAP) techniques. We identified 23 AFLP and 26, 7, and 5 MSAP outliers in *P. chienii*. Twenty‐one of the putative adaptive AFLP loci were found associated with climate and/or soil variables including precipitation, temperature, K, Fe, Zn, and Cu, whereas 21, 7, and 4 MSAP outliers were significantly related to precipitation of wettest month (Bio13), precipitation driest of month (Bio14), percent tree cover (PTC), and soil Fe, Mn, and Cu compositions. Total precipitation and precipitation in the driest seasons were the most influential factors for genetic and epigenetic variation, respectively. In addition, a high full‐methylation level and a strong correlation between genetic and epigenetic variation were detected in *P. chienii*. Climate is found of greater importance than soil in shaping adaptive (epi)genetic differentiation, and the synergistic effects of climate and climate–soil variables were also observed. The identified climate and soil variables should be considered when applying ex situ conservation.

## Graphical abstract

Whether conifers can withstand environmental changes especially temperature fluctuations has been controversial. Epigenetic analysis can provide new perspectives for solving the issue. An endangered gymnosperm species endemic to China, *Pseudotaxus chienii*, was selected as a representative to explore epigenetic architecture and further reveal the synergistic effects of climate and soil.

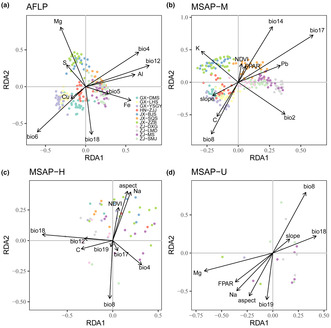

## 1 INTRODUCTION

Epigenetics emphasizes the potential of chemical tags on DNA (e.g., DNA methylation) to modulate gene expression in response to environmental stimuli without changes in the underlying DNA sequence (Jablonka & Raz, 2009; Richards, 2006; Verhoeven et al., 2016). It is also known that epigenetic information based on DNA methylation or chromatin modification could be stably inherited across generations (Heard & Martienssen, 2014; Verhoeven et al., 2010). In this context, investigation of epigenetic effects on evolutionary and ecological processes of natural populations has become a topic of great concern. Epigenetic phenomena occur relatively commonly in plants, and the involved mechanisms include DNA methylation, histone modification, microRNA, small interfering RNA, spatial location of DNA, and chromatin matrix or scaffold attachment regions (Carja & Feldman, 2012; Richards et al., 2010). In addition to genetic variation, epigenetic mechanisms can impose additional effects on gene expression and ultimately generate new phenotypic variants (Jablonka & Raz, 2009). For plants, epigenetic modifications induced by environmental stress cause not only significant phenotypic changes but also stable epigenetic inheritance to the next generation, possibly facilitating the adaptation to new environments (Dubin et al., 2015; Salmon et al., 2008). More importantly, they may also alter the action of natural selection on genetic variation (Richards et al., 2010). Hence, although epigenetic variation adds more complexity to evolutionary and ecological surveys at the population level, it is helpful to gain new insights for understanding population adaptation and evolutionary potential.

DNA methylation occurs widely in plants and is also currently best studied as a persistent epigenetic mark and best‐described epigenetic mechanism (Bird, 2007; Bossdorf et al., 2008; Herrera & Bazaga, 2010). It plays important roles in environmental stress responses in plants, especially long‐lived conifers with complex life cycles and genomes (Albaladejo et al., 2019; Gaspar et al., 2019; Herman & Sultan, 2016; Herrera et al., 2017; Platt et al., 2016; Shi et al., 2018; Verhoeven et al., 2016). Limited by long generation times, their allele frequency changes are very slow even under selection caused by rapid climate fluctuations (Bräutigam et al., 2013). Consequently, conifers must cope with environmental changes through phenotypic plasticity possibly related to epigenetic variation (Bräutigam et al., 2013; Li et al., 2018). In plants, DNA methylation occurs in different genomic contexts, such as CG, CHG, and CHH (H = A, T, or C; Gallego‐Bartolome, 2020; Meyer et al., 1994). Due to sensitivity to the environment, DNA methylation could be more involved in adaptive responses to changing environments via epimutations (Rey et al., 2020) and relatively more useful for genetically deficient or small and isolated populations (Bräutigam et al., 2013; Medrano et al., 2020; Rey et al., 2020). Epigenetic variation in the natural population can be investigated through methylation‐sensitive amplified polymorphism (MSAP; Richards et al., 2017). Even though MSAP is an anonymous molecular marker, it still provides a basis for the preliminary assessment of species epigenetic variation (Gaspar et al., 2019; Herrera & Bazaga, 2010; Li et al., 2018). In particular, MSAP‐based epigenetic variation can be compared with AFLP‐based genetic variation due to technical similarity, which is quite useful in distinguishing the contribution of genetic and epigenetic variations to adaptative evolution (Herrera & Bazaga, 2010).

In the context of global climate change, it becomes urgent to investigate plant population epigenetic variation. Climate warming is a major high‐profile issue, with an increase of 0.74°C in the global average temperature over the past hundred years, while the rate of warming has almost doubled in the last 50 years (IPCC, 2007). It is predicted that global temperatures will likely increase by 6°C in 2100 (Sommer et al., 2010), which will have a serious impact on the environment, especially producing stress on atmospheric water and soil. In fact, climate warming has become a major threat to plant diversity. It is speculated that up to half of all higher plants could go extinct due to global climate warming at the end of the century (Bramwell, 2007). Hence, it is important to elucidate which coping mechanisms plant populations may adopt in the face of environmental changes caused by climate warming. Currently, spatial study represents the most important research pathway, which suggests using contemporary populations with different locations (e.g., different latitudes or altitudes), different climatic or environmental factors, and different habitat types as surrogates that may emerge at different time points under the future climate warming scenario, to forecast population responses to climate change (Davis et al., 2005; Davis & Shaw, 2001; Feng & Du, 2022). In addition, exploring the population epigenetics of endangered plants also has conservation biological value, as knowledge of the relative importance of environmental factors to population differentiation is prerequisite to reducing the possibility of migrating individuals suffering from maladaptation, especially in ex situ conservation (Bekessy et al., 2003).

*Pseudotaxus chienii*, commonly known as “white‐berry yew,” is a dioecious evergreen conifer endemic to China. As a monotypic species in the genus *Pseudotaxus* (Taxaceae; Fu et al., 1999), its distinguishing feature lies in white aril and two white stomatal bands on the underside of leaves. The plant grows in subtropical mountain forests in gullies or on cliffs (Fu & Jin, 1992; Lin et al., 1999). The distribution range of *P. chienii* covers a large geographic area (large environmental variations), including montane regions of southern Zhejiang, southwestern Jiangxi, northwestern and southern Hunan, northern Guangdong, and northern Guangxi Zhuang Autonomous Region (Fu et al., 1999). Due to its low fertilization rate and poor natural regeneration, *P. chienii* has been listed separately as a second‐class nationally protected plant and a vulnerable species in the Chinese Red Data Book (Fu & Jin, 1992) and by the International Union for Conservation of Nature (IUCN; Thomas & Yang, 2013). Medium genetic diversity and putative adaptive genetic loci have been detected in the populations of *P. chienii* by using ISSRs (inter‐simple sequence repeats), SSRs (simple sequence repeats), and other molecular markers (Kou et al., 2020; Li et al., 2021; Liu et al., 2021; Su et al., 2009; Xu et al., 2020).

*Pseudotaxus chienii* has a wide geographic distribution, which may trigger epigenetic differentiation along environmental gradients, even possibly resulting in climatotypes or ecotypes with morphological and physiological differences associated with local adaptation (Rehfeldt et al., 2002; Wang & Yang, 2007). Conifers enable to maintain high intrapopulation genetic variation and low interpopulation differentiation due to longevity, high rates of pollen and seed flow, and outcrossing breeding (Hamrick, 2004). In addition, these patterns contribute to the rapid adaptation to environmental changes (Valcu et al., 2008). Research on climate‐related phenotypes, such as frost resistance, has shown that conifers indeed have high adaptive potential to extreme climatic events (Valcu et al., 2008). However, other studies have indicated that conifers are frail and sensitive to environmental changes, especially temperature fluctuations, which may exert strong negative effects on the growth, survival, and productivity of conifer populations (Rehfeldt et al., 1999, 2002). Hence, further analysis from an epigenetic perspective may provide more useful evidence for estimating the response of conifers to climate change (Eveno et al., 2008; Herrera & Bazaga, 2010; Jump et al., 2006; Li et al., 2014).

In this study, we used MSAP to investigate the epigenetic architecture of *P. chienii* with 314 individuals from 11 populations, and simultaneously used AFLP to explore genetic diversity as a control. Our specific objectives were (i) to assess population epigenetic compositions and test the evolutionary correlation between epigenetic and genetic variations at the population level; (ii) to examine the roles of climate/soil variables and their interactions on population epigenetic and genetic structures, aiming to identify the most important variables and determine the MSAP and AFLP loci by which climate/soil variables exert effects to drive epigenetic and genetic differentiation; and (iii) to disentangle the synergistic effects of climate and soil on population epigenetic differentiation (Bockheim et al., 2000). These investigations may be helpful in developing an efficient ex situ conservation strategy for *P. chienii* in the context of global changes.

## 2 MATERIALS AND METHODS

### 2.1 Population investigation and sample collection

**FIGURE 1:**
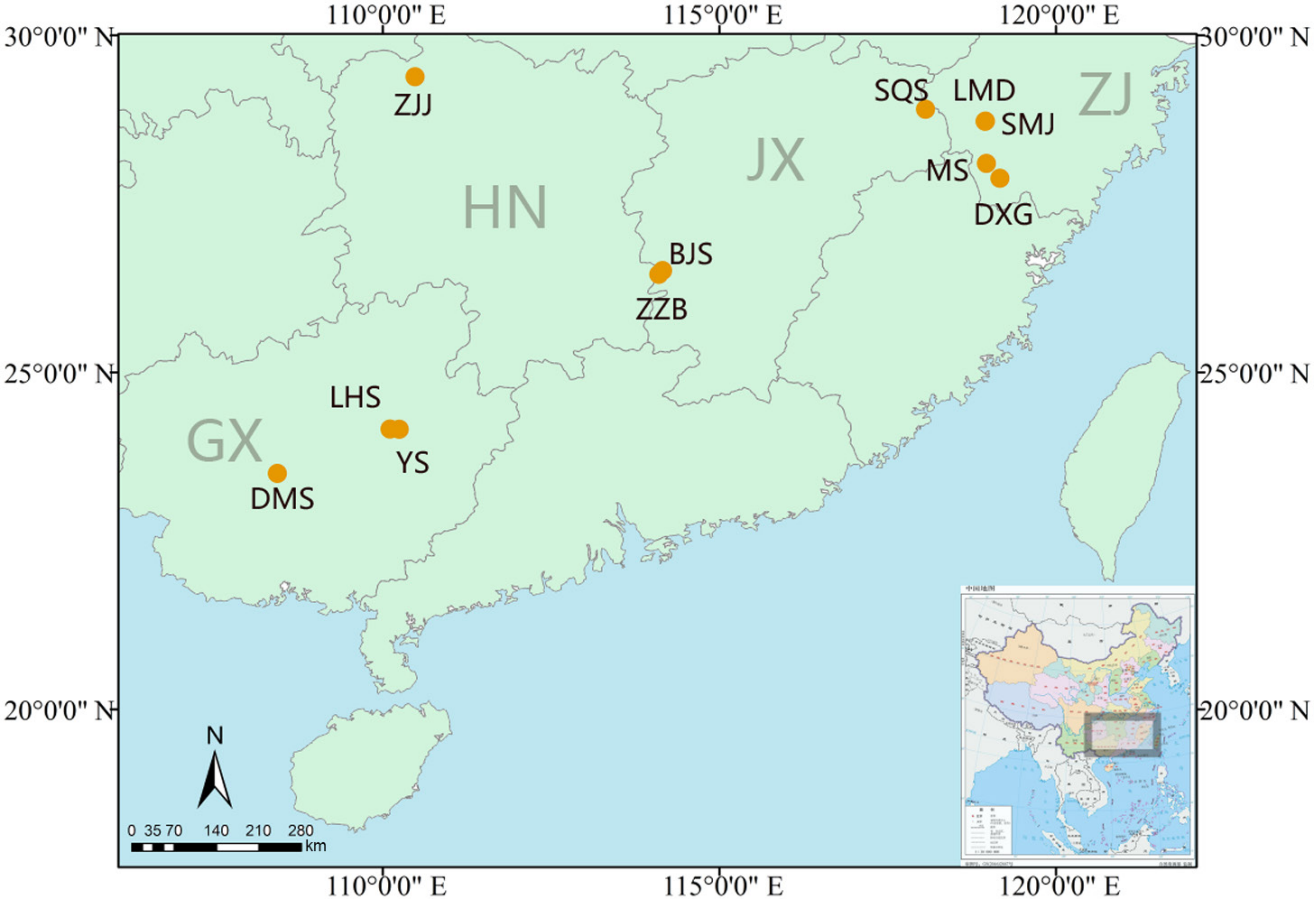
The 11 sampling sites across species distribution for *Pseudotaxus chienii* in China. Yellow dot represents the location of each population. ZJ, JX, HN, and GX denote Zhejiang Province, Jiangxi Province, Hunan Province, and the Guangxi Zhuang Autonomous Region, respectively. Population abbreviations, detailed locations, GPS coordinates, and sample sizes are shown in Table 1.

We sampled 312 individuals from 11 natural populations across the whole distribution range of *P. chienii* according to the specimen collection information of the Chinese Virtual Herbarium (CVH, http://www.cvh.org.cn/; Figure 1; Table 1). Voucher specimens (Qi Deng 2011012 SMJ1, LMD2, MS3, DXG4, SQS5, ZZB6, BJS7, ZJJ8, YSGY9, LHS10, and DMS 11) were deposited at the Herbarium of Sun Yat‐sen University, Guangzhou, China. Needle leaves were sampled and immediately preserved in silica gel after collection. Extraction of total genomic DNA was carried out using a modified cetyl trimethyl ammonium bromide (CTAB) method (Doyle & Doyle, 1987). In addition, 38 habitat soil samples of *P. chienii* populations were collected from the top 20‐cm layer after scraping off surface material (Table 1). Each soil sample was air‐dried, ground, and sieved (2‐ and 0.25‐mm screens for coarse and fine soil, respectively) for further chemical analysis.

**TABLE 1:** Location, GPS coordinate, sample size, and AFLP‐ and MSAP‐based diversity parameters for 11 populations of *Pseudotaxus chienii*.

Province	Population	Pop ID	Longitude	Latitude	Altitude	Individual size	Soil sample size	AFLP			MSAP‐M			MSAP‐H			MSAP‐U		
								*I*	*UHe*	*%P*	*I*	*UHe*	*%P*	*I*	*UHe*	*%P*	*I*	*UHe*	*%P*
Zhejiang (ZJ)	Shuimenjian	SMJ	118°57′32″ E	28°43′42″ N	830 m	30	4	0.304	0.197	75.51%	0.312	0.216	55.68%	0.161	0.095	55.71%	0.134	0.083	41.43%
	Longmending	LMD	118°57′13″ E	28°43′38″ N	1200 m	30	4	0.311	0.204	74.59%	0.294	0.202	55.71%	0.184	0.114	56.52%	0.157	0.101	42.82%
	Maoshan	MS	118°58′23″ E	28°06′08″ N	1120 m	30	4	0.283	0.185	68.84%	0.329	0.226	61.29%	0.160	0.094	57.91%	0.181	0.117	50.96%
	Daxiagu	DXG	119°10′24″ E	27°52′49″ N	1500 m	23	4	0.265	0.174	63.82%	0.270	0.186	51.24%	0.192	0.122	53.32%	0.122	0.078	33.94%
Mean	ZJ							0.291	0.190	70.69%	0.302	0.208	55.98%	0.174	0.106	55.87%	0.148	0.095	42.29%
Jiangxi (JX)	Sanqingshan	SQS	118°04′09″ E	28°54′19″ N	1500 m	30	3	0.293	0.191	73.48%	0.330	0.226	61.79%	0.197	0.122	60.68%	0.151	0.097	44.21%
	Zizhuba	ZZB	114°06′22″ E	26°27′18″ N	1300 m	30	3	0.255	0.166	64.06%	0.331	0.229	59.61%	0.180	0.109	58.75%	0.139	0.087	42.86%
	Bijiashan	BJS	114°09′41″ E	26°30′35″ N	1340 m	30	3	0.285	0.185	71.79%	0.259	0.178	50.25%	0.167	0.104	49.64%	0.145	0.094	39.12%
Mean	JX							0.278	0.181	69.77%	0.307	0.211	57.22%	0.182	0.112	56.36%	0.145	0.093	42.07%
Hunan (HN)	Zhangjiajie	ZJJ	110°28′56″ E	29°23′12″ N	1055 m	19	3	0.219	0.146	50.24%	0.305	0.214	54.19%	0.171	0.105	52.61%	0.134	0.087	35.68%
Guangxi Zhuang Autonomous Region (GX)	Yinshangongyuan	YSGY	110°14′53″ E	24°09′15″ N	1050 m	30	3	0.250	0.163	62.22%	0.285	0.196	52.03%	0.160	0.098	52.52%	0.126	0.080	38.25%
	Lianghuashan	LHS	110°06′47″ E	24°09′28″ N	1026 m	30	3	0.261	0.171	63.53%	0.220	0.150	41.47%	0.125	0.075	42.25%	0.113	0.072	33.03%
	Damingshan	DMS	108°26′12″ E	23°29′54″ N	1240 m	30	4	0.305	0.199	75.70%	0.214	0.147	40.14%	0.126	0.077	40.38%	0.096	0.062	26.59%
Mean	GX							0.272	0.178	67.15%	0.240	0.165	44.55%	0.137	0.083	45.05%	0.112	0.071	32.62%
Total								0.276	0.180	67.62%	0.286	0.197	53.04%	0.166	0.101	52.76%	0.136	0.087	38.99%

Abbreviations: *%P*, Percentage of polymorphisms; *I*, Shannon's information index; *UHe*, Unbiased expected heterozygosity.

### 2.2 AFLP analysis

We performed AFLP analyses for all samples according to the general method of Vos et al. (1995). Briefly, 500 ng of genomic DNA was digested by restriction enzymes *Eco*RI and *Mse*I and then ligated with adaptors. The 10‐fold diluted ligation was preamplified with *Eco*RI + A and *Mse*I + C primers. The preamplified product was diluted 1:50 with ddH_2_O and used as a template for selective PCR amplification to generate AFLPs. In total, 72 combinations of selective primers were screened. Finally, 10 combinations (AGC‐CAA/CAG/CTG, AGG‐CAG/CAC/CTG, AAC‐CAG/CTC, AAG‐CAG, and ACG‐CAG) with clear and polymorphic bands were chosen for genotyping (Table S1), of which the *Eco*RI selective primer was labeled with the fluorescent dye 6‐FAM (BGI). The final amplification products were separated by electrophoresis on an ABI PRISM 3130 sequencer (Applied Biosystems) with an internal size standard of ROX‐500. Electropherograms were automatically scored for presence (1) or absence (0) in a fragment range 50–500 bp using GeneMarker v2.2 (SoftGenetics, LLC; http://www.softgenetics.com/GeneMarker.php).

### 2.3 MSAP analysis

We conducted MSAP analysis using the same AFLP DNA template. *Hpa* II and *Msp* I were used in standard MSAP with recognition site 5′‐CCGG‐3′ (CG and CHG contexts). In this study, we adopted new isoschizomers, *Tfi* I and *Pfe* I, which can recognize 5′‐GAWTC‐3′ (CG, CHG, and CHH contexts; W = A/T; Xu et al., 2016). Except for the enzymes and corresponding NEB buffer, the new MSAP protocol is the same as the standard MSAP protocol (Xu et al., 2016). The DNA of each individual was digested using *Pfe* I/Bgl II and *TfiI/Bgl* II (New England Biolabs, NEB) in parallel reactions, which were further ligated to the *Bgl* II and *Tfi* I‐*Pfe* I adaptors (TaKaRa) using T4 DNA ligase (New England Biolabs, NEB). The digestion–ligation reaction was incubated for 10 h, followed by 15 min of inactivation. The ligated product was preamplified in 20 μL, including initial denaturation at 94°C for 5 min and 20 cycles with 94°C for 40 s, 56°C for 45 s, and 72°C for 1 min. The reaction mixture contained 2 μL 10 × buffer (with Mg^2+^), 1.5 μL dNTP (10 mM), 0.5 μL *Bgl* II preamplification primer (10 μM), 0.5 μL *Tfi* I/*Pfe* I preamplification primer (10 μM), 0.2 μL Taq DNA polymerase (5 U/μL), and 4 μL ligated product. The preamplified product was diluted 20‐fold as a template in subsequent selective amplification.

Similarly, selective amplification was performed in a 20 μL reaction mixture involving 2 μL 10 × buffer (with Mg^2+^), 1.5 μL dNTP (10 mM), 0.5 μL *Bgl* II‐N primers (10 μM), 0.5 μL *Tfi* I/*Pfe* I‐N primers (10 μM), 0.25 μL Taq DNA polymerase (5 U/μL), and 2.5 μL diluted preamplified product. The PCR conditions were as follows: an initial denaturation at 94°C for 5 min followed by 13 cycles of 94°C for 30 s, 65°C for 30 s, and 72°C for 1 min (decreasing 0.7°C per cycle), followed by 23 cycles of 94°C for 30 s, 56°C for 30 s, and 72°C for 1 min, and a final extension at 72°C for 5 min. The selective‐amplified products were visualized on an ABI3730XL capillary electrophoresis with LIZ1200 as an internal standard. The presence (1) and absence (0) were obtained using GeneMarker v2.2. We adopted 21 primer pairs generating clear polymorphic bands (Table S2).

### 2.4 Data analysis

Both AFLP and MSAP were used to assess the population variation and local adaptation of *P. chienii*.

#### 2.4.1 MSAP data scoring

Due to two cytosines in the double‐stranded *Tfi* I/*Pfe* I recognition site GAWTC, four types of bands were determined based on the presence (1) or absence (0) of amplicons in both *Bgl II/Tfi* I and *Bgl* II/*Pfe* I. Type I (0/0) was defined as full methylation with band absent in *Bgl* II/*Tfi* I and *Bgl* II/*Pfe* I. Type II (1/0) was referred to hemimethylation with band present in *Bgl* II/Tfi I and band absent in *Bgl* II/*Pfe* I. Type III (1/1) and Type IV were opposite to Type I and Type II as nonmethylation and uninformative loci, respectively. Cytosine methylation status can be obtained through differences in product combinations from *Bgl* II/*Tfi* I and *Bgl* II/*Pfe* I reactions. “Mixed scoring 2” can separate methylated markers with ^HMe^CG‐ or ^Me^CG‐sites and markers with ^HMe^CCG‐sites, respectively (Schulz et al., 2013). In this study, “Mixed scoring 2” was used to define MSAP‐M, MSAP‐H, and MSAP‐U (M, H, and U hereafter). For the M dataset, Type I (0/0) was scored as “1” and the other types as “0,” whereas Type II (1/0) was scored as “1” and the other types as “0” in the H dataset. In the U dataset, Type III (1/1) was scored as “1,” while the other types were scored as “0.” All subsequent analyses proceeded based on the four matrices.

#### 2.4.2 (Epi) genetic diversity and linkage disequilibrium analysis

Based on AFLP and MSAP datasets, we calculated genetic and epigenetic diversity parameters using GENALEX v6.41 (Peakall & Smouse, 2012), including Shannon diversity index (*I*), unbiased expected heterozygosity (*UHe*), and percentage of polymorphisms (*%P*). Using the *lmer* function of the R lme4 package (Bates et al., 2015), differences in *UHe* and *I* between populations or groups were tested based on a linear mixed‐effect model (LMM) with further detection of significance by using the *glht* function (Hothorn et al., 2008). The *Friedman.test* function was used to estimate the significance of the difference between genetic and epigenetic parameters of each dataset.

In addition, we estimated the linkage disequilibrium (LD) between pairs of molecular markers. The measure was calculated based on the index of association (*I*_*A*_; Brown et al., 1980) and the modified index of association (*rD*) between pairwise alleles by using the *ia* function of the R poppr package (Agapow & Burt, 2001) with 9999 permutations.

#### 2.4.3 (Epi) genetic differentiation and structure

Based on the M, H, U, and AFLP datasets, (epi)genetic differentiation among populations was calculated via AMOVA by using the *poppr amova* function of the R poppr package (Kamvar et al., 2014). Significance was tested by using the *randtest* function of ade4 with 9999 permutations. Moreover, we also used the *nei.dist* function of the R poppr package (Kamvar et al., 2014) to calculate the population pairwise Nei's (epi) genetic distance (Nei, 1972, 1978). To investigate (epi) genetic structure, we separately performed the unweighted pair‐group method with arithmetic means (UPGMA) using the *Aboot* function of the R poppr package, Bayesian clustering (STRUCTURE) by the website STRUCTURE HARVESTER (http://taylor0.BIOlogy.ucla.edu/STRUCTUREHarvester/; Earl & vonHoldt, 2012), and the discriminant analysis of principal components (DAPC; Jombart et al., 2010). The optimal number of clusters was obtained according to the highest ΔK value (Earl & vonHoldt, 2012). The STRUCTURE result was visualized through CLUMM (Jakobsson & Rosenberg, 2007) and DISTRUCT (Rosenberg, 2004). As for STRUCTURE analysis, we tested 21 clusters (*K* = 1–21). For each K, 20 runs were performed with a burn‐in length of 10,000 followed by 100,000 Markov chain Monte Carlo (MCMC) cycles. For DAPC, the *xvalDapc* function of the R adegenet package (Jombart, 2008) was used to conduct a cross‐validation to determine the optimal number of PCs, which was further employed in subsequent DAPC analysis using the *find.clusters* and *dapc* functions (Jombart et al., 2010). The pairwise *F*_st_ value was calculated using Arlequin software (Excoffier & Lischer, 2010).

#### 2.4.4 Correlation analysis between genetic and epigenetic variation

We first calculated dissimilarity indices in the AFLP, M, H, and U datasets using the *vegdist* function of the R vegan package (Oksanen et al., 2019). Based on the transformed datasets, we further applied the *Mantel* function to test the correlation between genetic and epigenetic variation by using Spearman's rank correlation with 999 permutations.

#### 2.4.5 Environmental variables

To evaluate the effects of environmental factors on population adaptation, we adopted 47 environmental variables, including 19 bioclimate, eight ecological and 20 soil variables.

The 19 climatic variables, including temperature and precipitation, during the period of 1950–2000 were obtained from WorldClim (http://www.worldclim.org; Hijmans et al., 2005) at a geographic grid resolution of 2.5 arc‐minutes (Tables S3 and S4).

Ecological factors included the normalized difference vegetation index (NDVI), enhanced vegetation index (EVI), leaf area index (LAI), fraction of absorbed photosynthetic active radiation (fPAR), and percent tree cover (PTC). All MODIS datasets were obtained from the Land Process Distributed Active Archive Center (LPDAAC, http://lpdaac.usgs.gov) for 2001–2018. The slope and aspect data were downloaded from the elevation data of WordClim v.1.4 and extracted using ArcMap 10.4 (Table S5). The altitude was recorded when sampling (Table 1).

The following 20 soil characteristics were measured: the water contents of fresh and air‐dried soil, the pH value and electrical conductivity (EC) of the soil suspension, and the contents of organic matter (OM), nitrogen (N), carbon (C), phosphorus (P), sulfur (S), silicon (Si), potassium (K), calcium (Ca), sodium (Na), magnesium (Mg), aluminum (Al), iron (Fe), manganese (Mn), zinc (Zn), copper (Cu), and lead (Pb). The chemical analysis procedure was as follows: first, the water contents of fresh and air‐dried soil were measured using 10‐g samples. Second, pH and electrical conductivity were determined in a solution of 2 g of coarse soil mixed with 8 mL of ultrapure water. Third, fine‐grained soils were used to perform the following analyses: (i) measuring the percentage of organic matter using potassium dichromate volumetry; (ii) determining the content of total carbon by using a total organic carbon analyzer (SHIMADZU) under burning conditions of 720°C; (iii) estimating the proportion of total nitrogen using a Kjeltec™ 8400 Analyzer unit (Foss) after digestion by using concentrated sulfuric acid at 190°C for 2 h; and (iv) assaying the concentrations of P, S, Si, K, Ca, Mg, Al, Fe, Mn, Zn, Na, Cu, and Pb by utilizing an inductively coupled plasma optical emission spectrometer (ICP–OES, PerkinElmer) after digestion with HNO_3_/HCl/HF (3/1/1, v/v/v) at MARS 6 Microwave Reaction System (CEM). Experimental treatments without soil samples were used as controls. All measurements were repeated three times (Table S6).

In view of the possible correlation between variables, we first used the *vifstep* function of the R usdm package to calculate the variance inflation in three levels, including bioclimate, ecology, and soil. Variables with VIF < 10 were retained.

#### 2.4.6 IBD and IBE

Based on the Pearson correlation coefficient, the *Mantel* function of the R vegan package (Oksanen et al., 2019) was used to test the correlation between Nei's genetic and epigenetic distance matrices and geography or environment (i.e., isolation by distance, IBD, and isolation by environment, IBE). The geographic distance was acquired from the pairwise Euclidean distance matrix of sampling locations. The environmental variables retained by VIF were transformed into a Euclidean distance matrix using the *dist* function in R. In addition, we used the *partial.mantel* function to test the independent relationship between epigenetic variation and environment or geography while controlling for genetic variation and the independent relationship between genetic variation and environment or geography when controlling for the epigenetic effect. We also evaluated the correlation between (epi)genetic and geography (environment) while controlling for the environment (geography).

Similar to the Mantel and partial Mantel tests, multiple matrix regression with randomization (MMRR) can also be used to estimate IBD and IBE. Its advantages lie in quantifying the effects of geographic and ecological isolation with no limitation to the number of distance matrices (Wang, 2013). We used MMRR to evaluate IBD and IBE, the response variables of which were Euclidian matrices transformed from (epi)genetic datasets, and the geographic and environmental distances were the same as those used in the Mantel test. MMRR was calculated by using the R script “MMRR” (Dryad database https://doi.org/10.5061/dryad.kt71r).

#### 2.4.7 Potential outlier detection

We used two approaches to detect adaptive loci with higher differentiation than the neutral loci. DFDIST (Beaumont & Nichols, 1996; Zhivotovsky, 1999) simulates a distribution of *F*_st_ values under neutral expectations to identify loci under positive selection from a plot of *F*_st_ against heterozygosity. First, the trimmed mean *F*_st_ (an estimate of the average “neutral” *F*_st_ value uninfluenced by outlier loci) was calculated after removing the 30% highest and lowest of the initial *F*_st_ observed in the empirical dataset. Second, based on 50,000 simulated loci, a null distribution of *F*_st_ values was generated with a mean *F*_st_ similar to the trimmed mean *F*_st_. Last, any loci outside the null distribution at 99.5% confidence levels were designated as potential outliers. DFDIST is conducted based on a symmetrical island model (Wright, 1951) with the assumption of Hardy–Weinberg equilibrium and drift–migration equilibrium (Bonin et al., 2006), which often provides false positives because it is unrealistic in most natural situations (Jones et al., 2013; Manel et al., 2009; Pérez‐Figueroa et al., 2010). To reduce false positives, we also used an alternative approach, BAYESCAN (Beaumont & Balding, 2004; Foll & Gaggiotti, 2008). Its advantages lie in the estimations of population‐specific *F*_st_ and the accommodations of different demographic histories between populations. Outliers may be detected by estimating a posterior probability for each locus with a reversible‐jump MCMC approach. The analysis parameters included a burn‐in of 50,000 iterations, a thinning interval of 20, and a sample size of 5000 (Pérez‐Figueroa et al., 2010; Wang et al., 2012). The “decisive” outliers were determined according to posterior odds (log_10_PO ≥ 2; Jeffreys, 1961).

#### 2.4.8 Correlation analysis based on GLMM

A generalized linear mixed model (GLMM) was used to test the associations between outliers and environmental variables. As an extension to GLMs (generalized linear models), the GLMM allows the analysis of nonnormal data while incorporating random effects (Lobreaux & Melodelima, 2015). Environmental variables are introduced as fixed effects, and groups are regarded as random effects. The analysis was performed by using the *glmer* function of the R lme4 package (Bates et al., 2015). Significance was assessed at the 95% and 99% confidence levels (CIs).

#### 2.4.9 Spatial variable

Population spatial (epi)genetic structure can be detected from environmental variables and spatial autocorrelation of populations using distance‐based Moran's eigenvector maps (dbMEM, originally termed “principal coordinates of neighbor matrices,” PCNM; Gibson & Moyle, 2020). The dbMEMs sketch a complicated spatial structure pattern that can be further adopted as a cofactor in subsequent redundancy analysis (Gibson & Moyle, 2020). In particular, dbMEM can model spatial structure at broad to fine scales (Abdo et al., 2013; Borcard & Legendre, 2002; Dray et al., 2006), which may influence the partitioning of genetic variation in different heterogeneous landscapes (Gibson & Moyle, 2020). In this study, 11 sampling locations were regarded as coordinates in the analysis. dbMEMs were calculated using the *pcnm* function in the R vegan package.

#### 2.4.10 Redundancy analysis

Redundancy analysis (RDA) is a constrained ordination approach to evaluate the explanatory power of multivariate predictors for multivariate responses (Gibson & Moyle, 2020). RDA can maximize the explained variance of the response variable. In this study, we employed RDA to distinguish the contributions of environmental and spatial variables to (epi)genetic variation.

To reduce collinearity, forward variable selection was applied to identify specific important environmental and spatial variables for generating selective differences across the range of *P. chienii*. These significant variables were first introduced to the model, followed by the remaining variables, until reaching stop criteria, which was the preselected alpha value of the significance level and the *R* ^2^ statistic of the global model (Blanchet et al., 2008). The *forward.sel* function of the R packfor package was used to carry out forward variable selection analysis (Dray et al., 2016), while the *rda* function of the R vegan package was used to perform RDA and partial redundancy analysis (pRDA) on retained variables and outliers for four datasets. When performing pRDA, geographic variables were treated as conditioned factors.

The *Varpart* function of the R vegan package (Oksanen et al., 2019) was used to distinguish the contributions of environmental and spatial covariates to (epi)genetic variation based on outliers and neutral loci. To avoid strong collinearity, we only adopted variables with VIF < 10. The analysis was performed based on four aspects: environmental variables alone (a), spatial structure alone (c), both environmental variables and spatial structure (a + b + c), and the residual effect (d). Significance was tested using the *annova.cca* function with 999 permutations (Borcard et al., 1992).

## 3 RESULTS

### 3.1 Environmental variables

The climate gradient throughout the natural geographic distribution of *P. chienii* is shown in Table S4, while the composition of the soil is displayed in Table S6.

### 3.2 DNA methylation level

**FIGURE 2:**
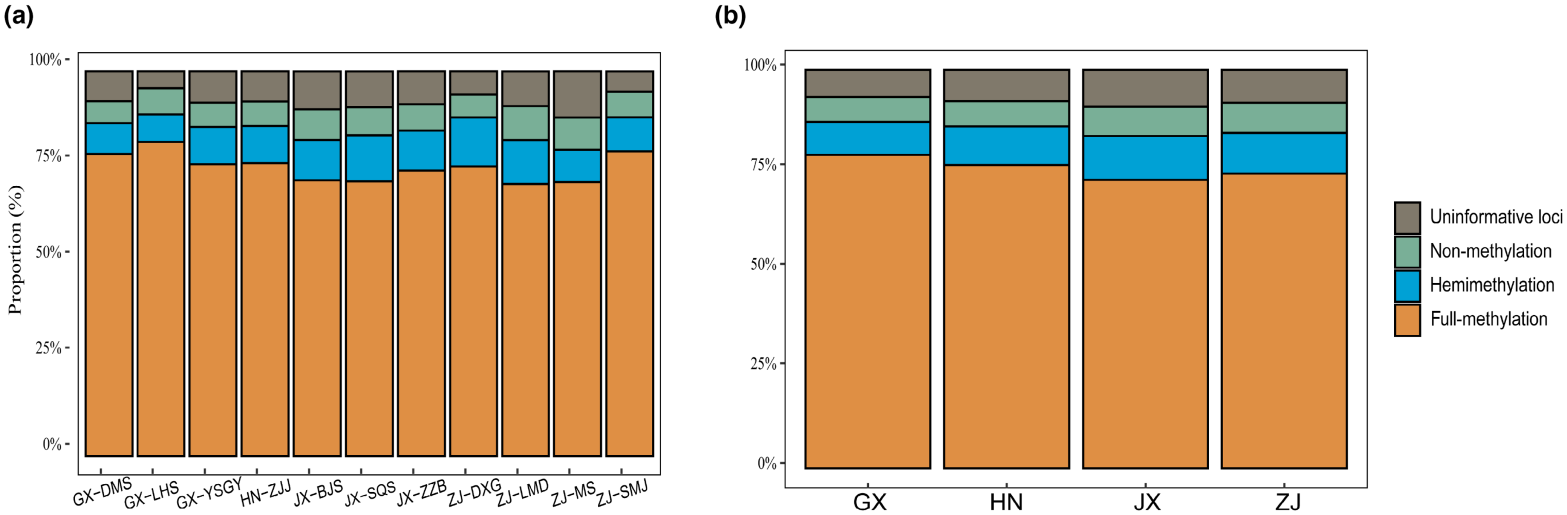
The methylation proportions at population (a) and province or region (b) level.

The different methylation statuses, including full methylation and hemimethylation, were investigated at the population and group levels. As a result, the full‐methylation level accounted for 70%, representing the highest percentage in populations. In addition, hemimethylation was slightly higher than nonmethylation (Table S7, Figure 2). For groups, populations in the Guangxi Zhuang Autonomous Region (GX) had the highest full‐methylation level, followed by populations in Hunan Province (HN), Zhejiang Province (ZJ), and Jiangxi Province (JX; GX: 78.70%, HN: 76.17%, ZJ: 75.04%, and JX: 74.24%). In contrast, the hemimethylation level was the highest in the Jiangxi population, followed by the Zhejiang, Hunan, and Guangxi populations, while the order of nonmethylation from high to low was ZJ, JX, HN, and GX populations.

### 3.3 (Epi)genetic diversity

For AFLP‐based genetic variation, 312 individuals yielded 2070 polymorphic loci. The average *I* across all populations was 0.276, ranging from 0.219 to 0.311, while the *UHe* ranged from 0.146 to 0.204 with an average of 0.180, and the overall *%P* was 67.7% with variation from 50.24% to 74.59%. The highest and lowest genetic diversity occurred in populations LMD and ZJJ, respectively (Table 1). LMM and pairwise comparisons analysis confirmed that the differences in *I* and *UHe* were significant between populations (*p* < .001).

For MSAP, we employed the “Mixed scoring 2” approach to obtain the MSAP‐M, MSAP‐H, and MSAP‐U datasets which generated 3154 (91%), 3098 (89%), and 2298 (66%) polymorphic epiloci, respectively. Corresponding to the above datasets, the average *I*, *UHe*, and *P%* and their variations are shown in Table 1. More importantly, the difference in *I* and *UHe* was not significant at the population (LMM test) and dataset levels (Friedman test). AFLP‐based genetic diversity was similar to MSAP‐M‐based epigenetic variation and higher than that of the other two epigenetic datasets (H and U). In addition, GX populations possessed the lowest epigenetic diversity, while relatively higher epigenetic diversity was found in JX and ZJ (Table 1; Table S8). Based on *I*_*A*_ and *rD*, LD was found among loci of AFLP (*I*_*A*_ = 10.741, *p* < .01; *rD* = 0.008, *p* < .01), M (*I*_*A*_ = 11.974, *p* < .01; *rD* = 0.0102, *p* < .01), H (*I*_*A*_ = 5.274, *p* < .01; *rD* = 0.0075, *p* < .01), and U (*I*_*A*_ = 6.301, *p* < .01; *rD* = 0.0116, *p* < .01) datasets across the populations (Table S8).

### 3.4 (Epi)genetic differentiation and STRUCTURE clustering

**FIGURE 3:**
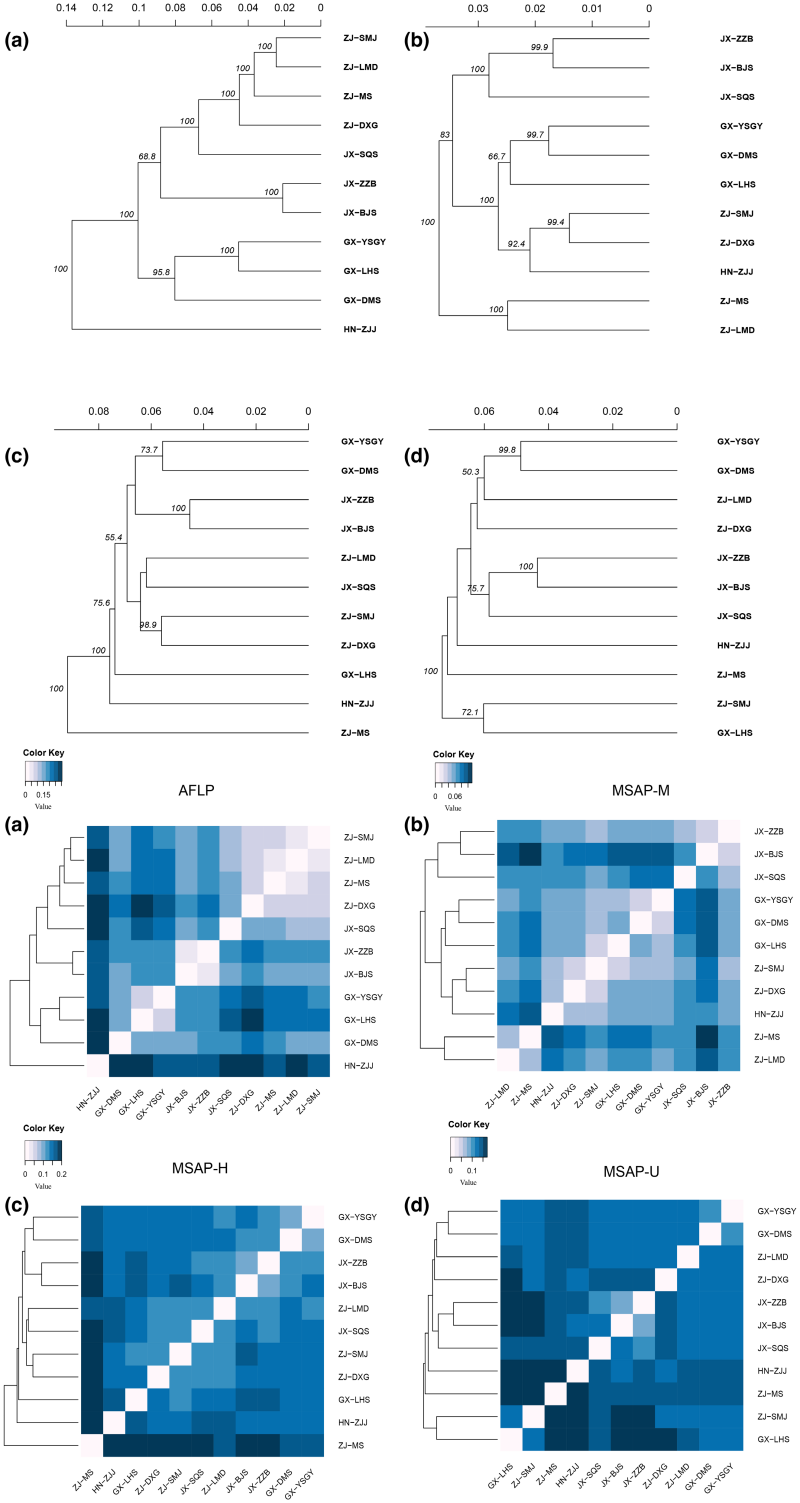
The UPGMA cluster analysis (above) and Nei's distance heatmap (below) based on AFLP (a), MSAP‐M (b), MSAP‐H (c), and MSAP‐U (d) datasets.

Based on AMOVA, the variation within populations greatly contributed to the (epi)genetic differentiation, which was 83.573%, 77.442%, 73.068%, and 76.301% in H, U, M, and AFLP, respectively (Table 2). For AFLP‐based UPGMA analysis, the clustered populations were consistent with their geographic locations except SQS. For the M dataset, population ZJJ was clustered in the ZJ populations, and GX populations formed a group. For the H and U datasets, no clear cluster was found corresponding to geographic locations (Figure 3).

**TABLE 2:** Analysis of molecular variance (AMOVA) based on AFLP and MSAP markers.

	Df	Φ‐Statistics	Var%
AFLP			
Among populations	10	0.237	23.700
Within populations	301		76.301
MSAP‐M			
Among populations	10	0.269	26.931
Within populations	303		73.068
MSAP‐H			
Among populations	10	0.164	16.427
Within populations	303		83.573
MSAP‐U			
Among populations	10	0.226	22.557
Within populations	303		77.442

Abbreviations: df, degree of freedom; Var%, percentage of variation.

*p* < .05.

**FIGURE 4:**
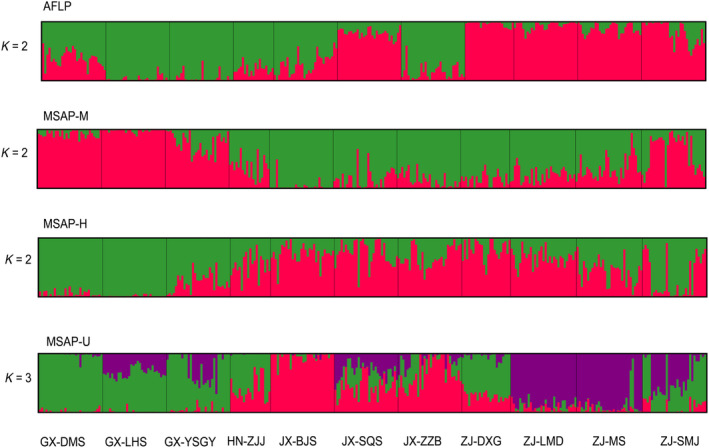
The genetic and epigenetic structure based on STRUCTURE. The optimal number of clusters is two, two, two, and three for AFLP, MSAP‐M, MSAP‐H, and MSAP‐U datasets, respectively.

**FIGURE 5:**
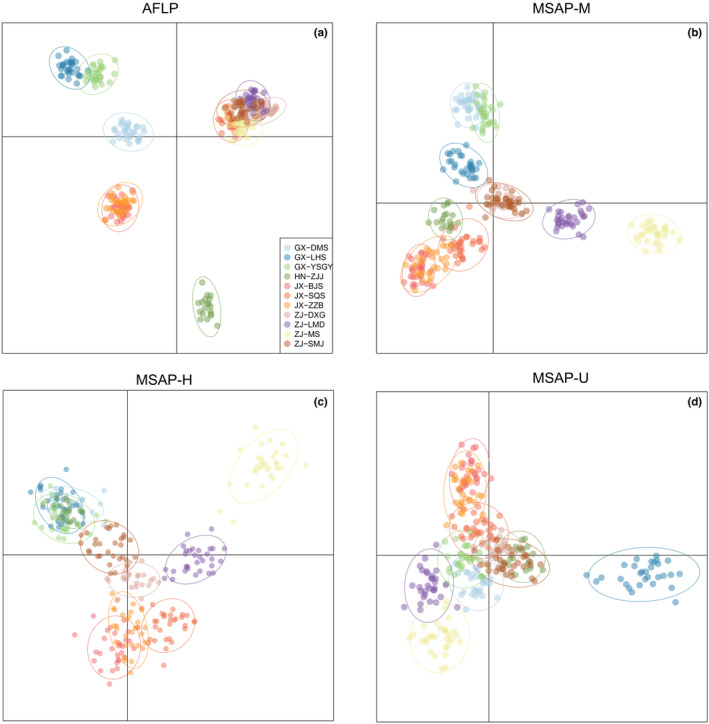
The genetic and epigenetic structures of *Pseudotaxus chienii* populations based on discriminant analysis of principal components (DAPC). (a) AFLP; (b) MSAP‐M; (c) MSAP‐H; (d) MSAP‐U.

In STRUCTURE clustering, the optimal *K* values were determined to be 2, 2, 2, and 3 in the AFLP, M, H, and U datasets, respectively. Based on AFLP, SQS and ZJ populations were clustered into a group, while the remaining other populations formed another group. In contrast, M, H, and U generated ambiguous clusters, which was similar to the UPGMA result (Figure 4). These results were further validated through DAPC analysis. Based on cross‐validation analysis, the numbers of retained PCs were 60, 100, 100, and 100, which explained 81.2%, 68.7%, 64.3%, and 66.6% of the total variance, respectively. The genetic structure showed that the populations were clustered into four groups corresponding to their geographic locations. In addition, the three epigenetic datasets generated similar patterns, indicating that two ZJ populations (MS and SMJ) did not cluster with other populations from the same location (Figure 5). The membership probability and pairwise *F*_st_ are shown in Figure S1 and Table S9.

### 3.5 The correlation between genetic and epigenetic variations

Based on the Mantel test, correlations on all pairwise comparisons showed that genetic and epigenetic variations exhibited significant correlation (*p* < .05). In addition, a significant correlation was also detected between epigenetic variations (*p <* .05; Table 3).

**TABLE 3:** The correlation coefficient between genetic and epigenetic variations based on Mantel test.

	AFLP	MSAP‐M	MSAP‐H
MSAP‐M	0.2739		
MSAP‐H	0.1448	0.8222	
MSAP‐U	0.2398	0.6141	0.3541

*p* < .05.

### 3.6 IBD and IBE

We adopted three types of environmental variables, including bioclimate, ecology, and soil factors. Based on VIF analysis, the retained variables were selected as Bio10, Bio11, Bio13, Bio14, PTC, LAI, fPAR, EVI, ALT, slope, aspect, K, Na, Fe, Mn, Zn, Cu, and Pb.

Mantel tests revealed a significantly positive correlation between Nei's genetic distance and geographic distance (*r* = .6890, *p* = .001) in the AFLP dataset, suggesting a strong “isolation by distance” pattern across populations. However, there was no correlation detected between geographic or environmental distance and epigenetic distance.

Based on partial Mantel tests (Table 4), epigenetic distance was found to be neither significantly related to environmental nor geographic distance when controlling for genetic distance. However, genetic variation was found significantly related to geographic and environmental distance when controlling for epigenetic distance. Further analysis of the relationship between (epi)genetic distance and geographic distance when controlling for environmental distance or vice versa showed that only genetic distance was related to geographic and environmental distance (Tables 4 and 5). In addition, MMRR analysis also showed that *P. chienii* had an IBD and IBE patterns (Table S10).

**TABLE 4:** Results of Mantel and partial Mantel test.

Dataset	Geographical matrix						Environmental matrix					
	Mantel test		Partial Mantel test (environment)		Partial Mantel test (genetic)		Mantel test		Partial Mantel test (environment)		Partial Mantel test (genetic)	
	Correlation coefficient	*p*	Correlation coefficient	*p*	Correlation coefficient	*p*	Correlation coefficient	*p*	Correlation coefficient	*p*	Correlation coefficient	*p*
AFLP	.598	.001	.500	.001			.568	.002	.457	.003		
MSAP‐M	.165	.100	.180	.063	.1545	.187	−.002	.465	−.072	.641	−.051	.599
MSAP‐H	.123	.147	.104	.172	.042	.416	.136	.248	.087	.297	.035	.35
MSAP‐U	.153	.119	.121	.172	−.031	.582	.110	.260	.056	.397	−.074	.657

**TABLE 5:** Top‐level environmental variables and their contribution to AFLP and MSAP variation determined by forward selection with RDA.

	*R* ^2^	*R* ^2^Cum		*R* ^2^	*R* ^2^Cum
**AFLP**			**MSAP‐M**		
Bio12	0.233	0.230	Bio17	0.199	0.196
Mg	0.119	0.348	Bio14	0.172	0.367
Bio4	0.085	0.431	C	0.052	0.417
Bio18	0.073	0.503	NDVI	0.057	0.472
Al	0.034	0.535	fPAR	0.029	0.501
Cu	0.028	0.562	K	0.031	0.531
Fe	0.019	0.580	Bio8	0.025	0.555
Bio6	0.014	0.593	Bio2	0.021	0.575
S	0.011	0.603	Slope	0.017	0.591
Bio5	0.006	0.608	Pb	0.011	0.601
**MSAP‐H**			**MSAP‐U**		
Bio18	0.102	0.099	Mg	0.133	0.130
Bio8	0.083	0.180	Na	0.137	0.265
Bio17	0.079	0.257	Bio8	0.072	0.335
C	0.060	0.315	Slope	0.064	0.398
Bio4	0.037	0.350	Bio19	0.048	0.445
NDVI	0.029	0.377	Aspect	0.041	0.485
Bio12	0.023	0.399	fPAR	0.027	0.510
Bio19	0.011	0.408	Bio18	0.021	0.530
Aspect	0.009	0.416			
Na	0.006	0.420			

Abbreviations: *R* ^2^, the contribution of variables to genetic and epigenetic variances; *R* ^2^Cum, the cumulative *R* ^2^ of the selected variables.

*p* < .001.

### 3.7 Detection of potential outliers

We simultaneously identified 23 outliers under selection in AFLPs through DFDIST with a confidence level of 99.5% and BAYESCAN at a threshold of log_10_PO ≥ 2.0 (posterior probabilities higher than 99%; Figure S2). Similarly, 26, 7, and 5 outliers were separately detected for the M, H, and U datasets using the same two programs, respectively (Figure S2).

### 3.8 Environmental correlation analysis based on GLMM

Based on GLMM analysis, correlations were found between environmental variables; and 21 and 22 outliers from the M and AFLP datasets, whereas 7 and 4 outliers from the H and U datasets were detected significantly related to environmental variables, respectively. Soil factors including K, Fe, Cu, and Zn were found related to outliers across the datasets. Bioclimatic variables (Bio13, Bio14, and Bio16) were also found to correlate with the outliers (Table S11).

### 3.9 Spatial data and redundancy analysis

We used redundancy analysis (RDA) to quantify the relationship of (epi)genetic variation with environmental variables. The 11 sampling locations were used to generate seven eigenvectors with positive eigenvalues, which represented a positive spatial autocorrelation between populations. These eigenvectors were regarded as spatial predictors (PCNM1‐7; Table S12).

**FIGURE 6:**
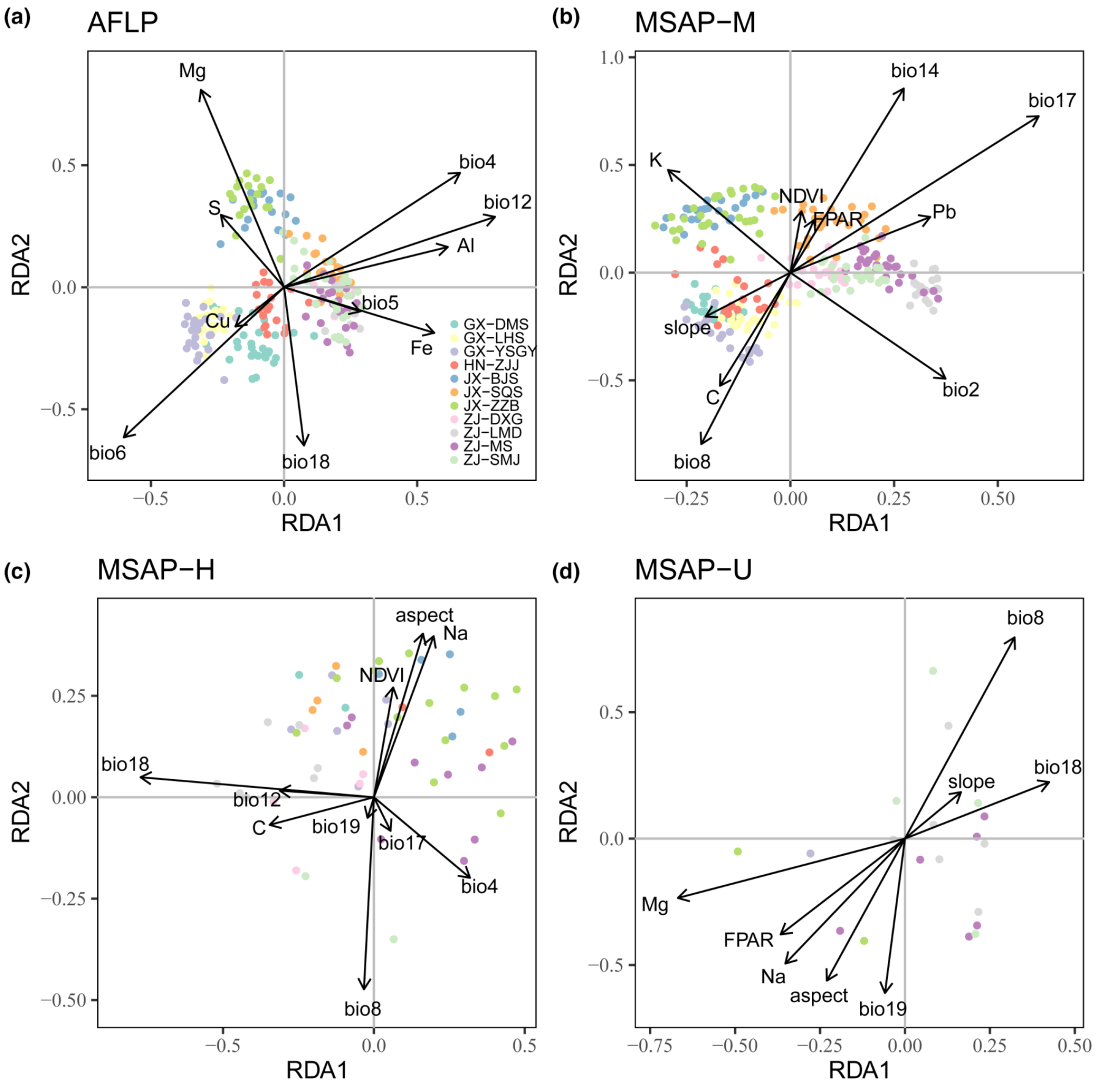
Significant associations between environmental variables and adaptive loci in redundancy analysis (RDA). Black arrows represent the environmental variables from forward selection. Colorful dots refer to individuals of different populations. (a, b, c, and d) correspond to AFLP, MSAP‐M, MSAP‐H, and MSAP‐U datasets, respectively.

Based on forward selection with RDA, Bio12 was identified as the most significant variable affecting genetic variation, while Bio17, Bio18, and Mg were the most important variables based on the M, H, and U datasets, respectively. In addition, all environmental variables used in forward selection contained Bio8 in epigenetic datasets, indicating a crucial role of Bio8 in epigenetic variance (Table 5; Figure 6). Moreover, pRDA was also conducted by treating geographic variables as conditioned factors, which generated very similar results to RDA (Du et al., 2020).

**FIGURE 7:**
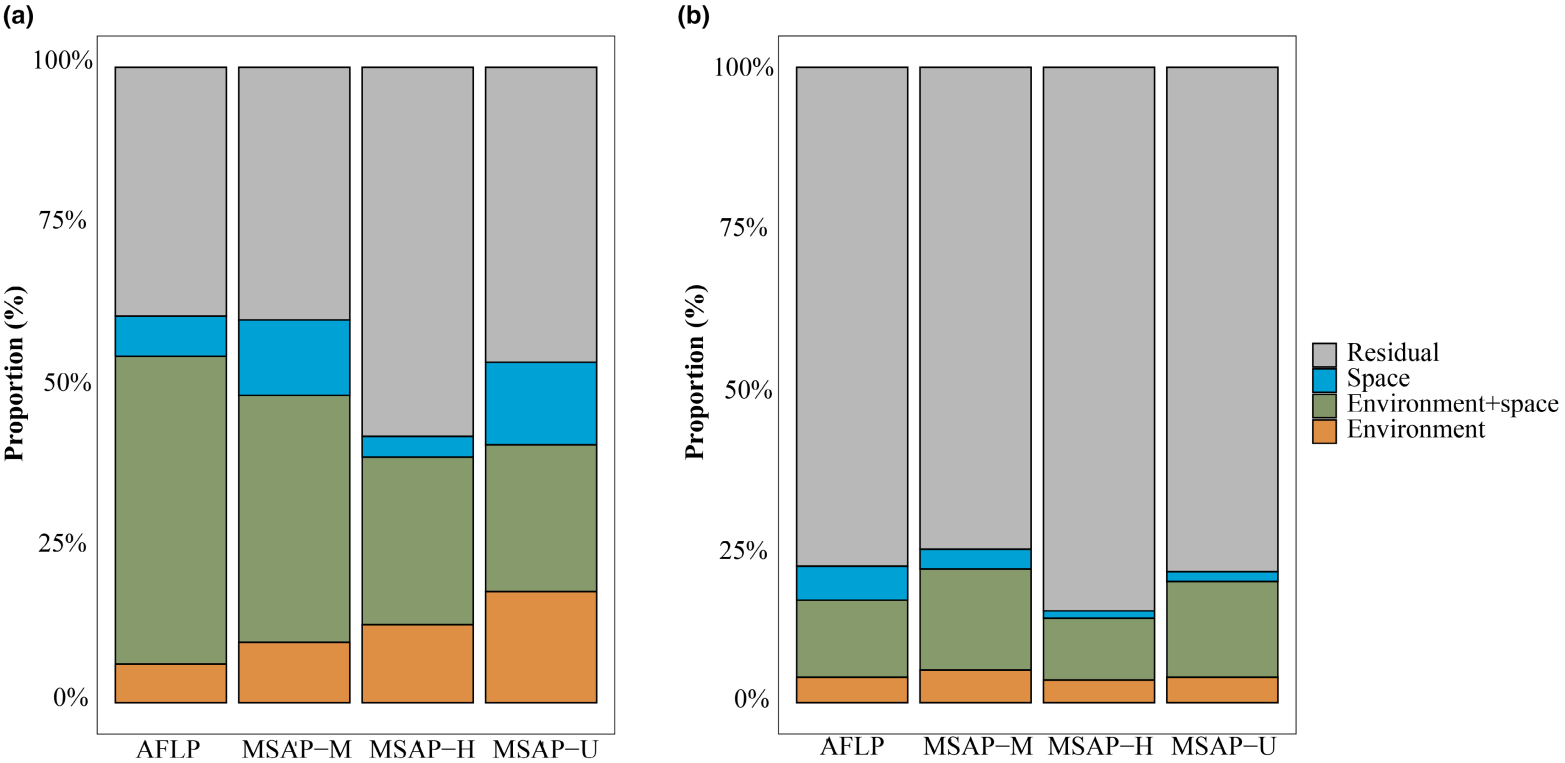
Variance partitioning between environment and space based on outlier (a) and neutral loci (b) derived from AFLP, MSAP‐M, MSAP‐H, and MSAP‐U datasets, respectively. Orange, green, and blue colors indicate the variance explained by environment, both environment and space, and solely space, respectively.

Environmental and spatial variables with VIF < 10 were further used for variance partitioning. The results derived from the *vapart* function showed that the portion of genetic variance explained by environmental variables alone (a), spatial patterns alone (c), and their joint effect (b) accounted for 6.08%, 6.32%, and 60.84%, respectively. For the epigenetic variance, environment alone accounted for 9.51%, 12.26%, and 17.47% based on M, H, and U, respectively, while space correspondingly accounted for 11.87%, 3.28%, and 13.01%, respectively; and the joint effect of environment and space separately accounted for 60.24%, 41.92%, and 53.58% of the total variance in the three datasets, respectively. The results implied that 50% of the total amount of variance could be explained by both environment and space. For genetic variance, environmental variables alone could explain slightly more than spatial ones. For epigenetic variance, the explanation of environment alone increased from M to H to U, whereas the explanation of space from high to low was U, M, and H. In addition, more variance was ascribed to environment rather than space except for the M dataset‐derived outliers (Figure 7a).

## 4 DISCUSSION

This study first provided a detailed assessment of epigenetic architecture for *P. chienii* populations based on MSAP. Currently, MSAP is widely used to investigate the methylation level of nonmodel organisms, especially for long‐lived conifers without genome information (Avramidou et al., 2015). In this study, the MSAP results showed that the level of full methylation (75.04% on average) was higher than that of hemimethylation or nonmethylation in *P. chienii*. The total methylation level was up to 84.97%, which was higher than that in other conifers (e.g., *Pinus pinea*; Sáez‐Laguna et al., 2014) and angiosperms (e.g., *Oryza sativa*, 16.3%, Xiong et al., 1999; and *Arabidopsis thaliana*, 5.8%, Kawakatsu et al., 2016).

The DNA methylation level is related to multiple factors. It firstly depends on organs or developmental stages, especially from the juvenile to adult phase (Huang et al., 2012). For instance, adult vegetative shoots have higher DNA methylation than juvenile and rejuvenated shoots (8% vs. 6.5%–7.5%, respectively) in *Sequoia sempervirens* (Huang et al., 2012), and a similar phenomenon has also been noted in *Pinus radiata* (Fraga et al., 2002). In the present study, our DNA samples were acquired from mature leaves at the same developmental stage. High DNA methylation levels may also be associated with abundant transposable elements and pseudogenes and genome hypermethylation (Ausin et al., 2016; Sáez‐Laguna et al., 2014). In addition, this study may underestimate the genome‐wide DNA methylation level in *P. chienii* because *Tfi* I/*Pfe* I was used as a methylation restriction enzyme with the recognition site 5′‐GAWTC‐3′ rather than conventional *Eco*R I*/Hpa* II with the recognition site 5′‐CCGG‐3′ (Xu et al., 2016).

This study also examined the AFLP‐based genetic diversity. Similar to ISSR and SSR (Su et al., 2009; Xu et al., 2020), we found that *P. chienii* had medium AFLP‐based genetic diversity compared to other plants analyzed with the same marker, including conifers and angiosperms, such as *P. tabulaeformis*, *P. asperata*, *P. monticola*, *Larix principis‐rupprechtii*, *Taiwania cryptomerioides*, *Prunus mume*, *Plantago lanceolata*, and *Vitex negundo* var. *heterophylla* (Di et al., 2014; Di & Wang, 2013; Gaspar et al., 2019; Kim et al., 2011; Li et al., 2018; Liu et al., 2018; Ma et al., 2018; Xue et al., 2005). It has been noted that the diversification and shift of conifers may be correlated with the cooler and more arid conditions after the late Eocene (Brodribb et al., 2014; Stull et al., 2021). Importantly, environmental heterogeneity associated with the broad range size may play important role in their diversification as well. When genetic diversity is insufficient to protect against harsh environments, epigenetic variation may act as compensation (Richards, 2006). In this study, the ZJJ population in Hunan was found to have the lowest genetic variation but the highest epigenetic variation, second only to the Jiangxi population (Table 1). We speculate that epigenetic variation could improve the adaptability of populations by compensating for the lack of genetic variation, as suggested by previous studies in plants such as *Plantago lanceolata* and *Alternanthera philoxeroide*s (Gao et al., 2010; Gaspar et al., 2019).

There exist three types of possible correlation between genetic and epigenetic variations: obligatory, facilitated, and pure correlations (Foust et al., 2016). In obligatory epigenetic variation, epigenotype is completely controlled by genotype; while in facilitated epigenetic variation, epigenotype depends on both genotype and environmental factors. By contrast, pure epigenetic variation is entirely independent of genetic variation (Foust et al., 2016). However, it is hard to determine whether the patterns at specific epiloci are merely a downstream product of genetic variation or independent of genetic variation without validation by sequencing. Even if detailed genomic sequence data are available, it remains impossible to collect all methylated and unmethylated genes, and it is unclear how functional epialleles assist organisms in coping with actual complex environmental stress (Foust et al., 2016). Moreover, it is also difficult to disentangle the effects of genetics, epigenetics, and the environment in woody plants without common garden experiments (Foust et al., 2016). MSAP markers can only detect epigenetic structures that are not explained by genetics in natural populations. In this study, *P. chienii* exhibited AFLP‐based genetic IBD and IBE patterns, as revealed by other molecular genetic markers (Kou et al., 2020; Li et al., 2021; Su et al., 2009). However, it lacked epigenetic IBD and IBE patterns, showing a mixed population epigenetic structure. Notably, a significant correlation was found in *P. chienii* populations between genetic and epigenetic variations and between methylated and unmethylated datasets. The results implied that their epigenetic variation was not independent of genetic variation. A similar phenomenon was present in *Viola elatior* and *T. cryptomerioides* (Li et al., 2018; Schulz et al., 2014).

Next, we observed that the epigenetic structure of *P. chienii* populations presented a close clustering of populations from different geographical locations with high gene flow. In addition, nuanced differences were found in epigenetic structure derived from different datasets, possibly being related to the difference in enzyme specificities to methylation and functional association with CG versus CHG in plants (Foust et al., 2016). Notably, epigenetic divergence was found between the MS and LMD populations and other Zhejiang populations. Kou et al. (2020) indicated that *P. chienii* in Zhejiang possibly has undergone a stronger divergent selection in comparison to other regions. Local environmental conditions may have facilitated the epigenetic divergence (Herrera et al., 2017).

Outliers refer to loci under selective pressures and of vital ecological significance (Richards et al., 2010). In this study, 23 AFLP loci potentially under selection were identified by running DFDIST and BAYESCAN. The proportion of outliers (1.3%) detected in *P. chienii* was much smaller than the average value for most plant species (8.9%; Strasburg et al., 2012). Nevertheless, the proportion is quite similar to the results found in other conifers, including *P. taeda* (1.3%; Eckert et al., 2010), *Cryptomeria japonica* (1.4%; Tsumura et al., 2012), and *Picea mariana* (1.7%; Prunier et al., 2012). The detection of 1.3% putative adaptive AFLP loci implied that there are maybe a relatively small number of genetic loci involved in the adaptation of *P. chienii* (Wakamiya et al., 1993; Wang et al., 2012). Compared with angiosperms, conifer genomes possess a higher recombination rate (Brown et al., 2004), lower proportion of coding regions (Wang & Ran, 2014), and much slower (approximately 15 times) nucleotide substitution rates (Buschiazzo et al., 2012). These factors may affect the detection of putative adaptive loci as well (Namroud et al., 2008).

Of epigenetic analysis, the maximum number (26) of MSAP outliers was found in the M dataset, whereas only seven and five MSAP outliers were detected in the H and U datasets, respectively. In comparison to another conifer *T. cryptomerioides* (Li et al., 2018), relatively high proportion of epi‐adaptive loci were detected in *P. chienii*. Considering 23 outliers identified in the AFLP dataset and 26 MSAP outliers in the M dataset, it seems epigenetic variation may be of similar importance as genetic variation for the adaptive evolution of *P. chienii*.

Of the 23 putative adaptive AFLP loci, 21 were found to correlate with climate or climate and soil variables. This result indicates a relatively greater importance of climate factors than factors linked to soils (see below) in shaping genetic differentiation (Manel et al., 2009). In particular, no AFLP adaptive loci that are solely linked to temperature were detected; in comparison, seven adaptive AFLP loci that are specifically related to precipitation and 10 AFLP adaptive loci that are simultaneously associated with both temperature and precipitation were identified. Similar results have been obtained in conifers such as black spruce (*P. mariana*; Prunier et al., 2012) and European larch (*Larix decidua*; Mosca et al., 2012). These findings suggest that the synergistic effect between temperature and precipitation might be more important than their separate action in causing genetic differentiation at the adaptive loci. It has indeed been shown that transcriptional networks responsive to dehydration and cold stresses are interconnected in *Arabidopsis* (Yamaguchi‐Shinozaki & Shinozaki, 2006).

Correlation analysis based on a generalized linear model found that the most influential factor was precipitation of driest quarter (Bio17), followed by precipitation of warmest quarter (Bio18), and soil Mg^2+^ in the M, H, and U datasets. The results show that precipitation plays an important role in driving the divergence of *P. chienii*, which was possibly related to its preference for rainfall (Li et al., 2021; Liu et al., 2021). Due to their sessile lifestyle, plants develop epigenetic variation to cope with environmental changes and improve potential adaptation (Richards et al., 2010). Further surveying also found that more epigenetic loci (M dataset) were correlated with precipitation of driest quarter (Bio17) compared to genetic variation. As a long‐lived conifer with a preference for wet environments, *P. chienii* accumulates more epigenetic variation with fluctuations in dry and wet environments, especially when the temperature increases. Current research shows that high‐temperature stress significantly induces upregulation of the full‐methylation rate in *Pinellia ternate* (Chao et al., 2020). This phenomenon also appears in other water‐deficient plants (Gourcilleau et al., 2010; Labra et al., 2002; Pagel et al., 2020).

The natural populations of *P. chienii* have a wide‐range distribution, from 21° N to 29° N and 108° E to 121° E (Fu et al., 1999). Chen et al. (2013) noted that *P. chienii* undergoes climatic fluctuations in its distribution range from east to west as drastic as subtropical monsoon climate, eastern Asia humid monsoon climate, south subtropical mountain humid monsoon climate, and subtropical mountain humid monsoon climate. There are thus significant differences in the temperature and precipitation variables affecting the geographic areas examined in our study, especially in terms of isothermality (Bio3), minimum temperature of coldest month (Bio6), precipitation seasonality (Bio15), precipitation of driest quarter (Bio17), and precipitation of coldest quarter (Bio19; Table S4). *Pseudotaxus chienii* was estimated to have arisen at least 120 million years ago (Cheng et al., 2000). It has undergone considerable changes in climate ever since, including several glacial and interglacial periods, and has consequently developed adaptation to climatic differences. In line with this, Wang and Yang (2007) documented the adaptive phenotypic variation in *P. chienii* generated by the spatial heterogeneity of climate.

The present study demonstrated that the majority of genetic and epigenetic variation (60.84% and 53.58%, respectively) was explained by both environmental and spatial components. Liu et al. (2021) found that temperature and precipitation are important drivers of adaptive evolution in *P. chienii*. Here, other environmental variables were also found relative to both (epi)genetic and spatial structure. Specifically, soil K, Zn, Fe, and Cu were correlated with the putative outliers,

Potassium (K) and iron (Fe) are essential macronutrients for plants, fulfilling important functions in metabolism, growth, and stress adaptation (Morrissey & Guerinot, 2009; Nieves‐Cordones et al., 2012). Copper (Cu) is not only an essential micronutrient for plants but also a two‐sided element. Copper deficiency impacts the function of key cell enzymes, whereas Cu overload may generate highly reactive oxygen species (ROS) that cause severe cell damage (Adamo et al., 2012). Zinc (Zn) is involved in auxin synthesis, signal transduction, and transcriptional regulation and is an important component of zinc finger proteins and approximately 300 enzymes (Epple et al., 2003; Ordiz et al., 2002; Riechmann et al., 2000). Yang (2012) has previously pointed out that a low Zn concentration promotes, whereas a high concentration inhibits the germination and growth of new branches of *Taxus media*, which is closely related to *P. chienii*.

A moderate‐to‐high genetic and epigenetic variation and high (epi)genetic differentiation were identified in *P. chienii* compared to other conifers (Table 6). Given the population (epi)genetic data, together with the survey showing that 98% of the *P. chienii* individuals survive in Zhejiang (Hu et al., 2003), these studies highlight the priority to conserve the small and localized populations outside Zhejiang. Although *P. chienii* exhibits no depletion of genetic variation, epigenetic variation could still promote adaptation to changing climatic and soil conditions since its long generation time, insufficient pollination, and limited seed dispersal may inevitably constrain the ability to migrate and track suitable habitats (Aitken et al., 2008; Davis & Shaw, 2001). Such life history characteristics must be considered when defining a conservation strategy. Based on the associations revealed between the putative adaptive (epi)loci and climatic and soil variables, special attention should be given to factors including temperature, precipitation, K, Fe, Zn, and Cu to perform ex situ conservation.

**TABLE 6:** Genetic and epigenetic diversity and population differentiation obtained from different gymnosperm species.

Species	*UH*_e_	PPL	*I*	*F*_st_	References
AFLP					
*Abies ziyuanensis*	0.136	35.73%	0.200	0.482	Tang et al. (2008)
*Pinus tabulaeformis*	0.216	85.17%	0.219	0.304	Di and Wang (2013)
*Pseudotaxus chienii*	0.180	67.62%	0.276	0.237	This study
*Picea asperata*	0.156	67.62%	0.227	0.231	Xue et al. (2005)
*Pinus monticola*	0.235	90.20%	0.353	0.201	Kim et al. (2011)
*Larix principis‐rupprechtii*	0.225	71.90%	0.341	0.194	Di et al. (2014)
*Taiwania cryptomerioides*	0.211	58.90%		0.0783	Li et al. (2018)
MSAP‐M					
*Taiwania cryptomerioides*	0.151	55.7%		0.0134	Li et al. (2018)
*Pseudotaxus chienii*	0.197	53.04%	0.286	0.269	This study
MSAP‐U					
*Taiwania cryptomerioides*	0.162	46.5%		0.0947	Li et al. (2018)
*Pseudotaxus chienii*	0.087	38.99%	0.136	0.226	This study
MSAP‐H					
*Pseudotaxus chienii*	0.101	52.76%	0.166	0.164	This study

Abbreviations: *F*_st_ represents genetic differentiation among populations; *I* refers to Shannon's information index; PPL is percentage of polymorphic loci; *UH*_*e*_ represents Nei's genetic diversity.

In addition, our results also show implications for the endangering mechanism of *P. chienii*. Su et al. (2018, 2020) have noted that the decrease in natural populations of *Taxus cuspidata* (Taxaceae), a closely related species of *P. chienii*, is mainly caused by recent fragmentation rather than historical climatic oscillations. In line with this, no lack of genetic and epigenetic variations was detected in the small populations of *P. chienii* in the present study. This raises the possibility that recent anthropogenic disturbance may also play a major role in the endangerment of *P. chienii*, which reserves further investigation.

## 5 CONCLUSION

Based on MSAP, we first revealed the epigenetic variation pattern of *P. chienii* populations, which was found significantly associated with climate and soil factors. A similar phenomenon was observed in its AFLP‐based genetic variation. We identified 23 AFLP and 26, 7, and 5 MSAP outliers (corresponding to the M, H, and U datasets, respectively) as putative adaptive (epi)loci in *P. chienii*. Twenty‐two of the putative adaptive AFLP loci, and 21, 7, and 4 putative MSAP outliers (corresponding to the M, H, and U datasets, respectively) were found associated with climate and/or soil K, Fe, Zn, and Cu contents. Our results highlight the synergistic effects between environmental factors in (epi)genetic adaptation. Under ongoing global warming, populations of *P. chienii* are predicted to shrink in southern China and shift to central China (Li et al., 2021; Liu et al., 2021). The climate and soil factors identified here should be considered when applying ex situ conservation.

## AUTHOR CONTRIBUTIONS

**Yingjuan Su:** Funding acquisition (equal); project administration (equal); supervision (equal); writing – review and editing (equal). **Li Liu:** Data curation (equal); methodology (equal). **Qi Deng:** Data curation (equal); methodology (equal). **Zhuyan Lü:** Formal analysis (equal); writing – original draft (equal). **Zhen Wang:** Formal analysis (equal); writing – review and editing (equal). **Zhiqing He:** Formal analysis (equal). **Ting Wang:** Funding acquisition (equal); project administration (equal); supervision (equal); writing – review and editing (equal).

## FUNDING INFORMATION

This work was supported by the National Natural Science Foundation of China (31872670 and 32071781), Guangdong Basic and Applied Basic Research Foundation (2021A1515010911), Science and Technology Projects in Guangzhou (202206010107), and Project of Department of Science and Technology of Shenzhen City, Guangdong, China (JCYJ20190813172001780 and JCYJ20210324141000001), Science and Technology Base and Talent Special Project of Guangxi (AD19110088), and Basic Ability Improvement Project for Young and Middle‐aged Teachers in Universities of Guangxi (2018KY0334).

## CONFLICT OF INTEREST STATEMENT

The authors declare no conflicts of interest.

## Supporting information


Appendix S1:

## Data Availability

The datasets used for this study are available through Dryad at the time of publication (https://doi.org/10.5061/dryad.m0cfxpp8q). Currently, the data can be downloaded at: https://datadryad.org/stash/share/mR3sxqPBxmydoFeydoF4‐THWt4oHqNwDSatHdleOp9U. Once the manuscript is accepted, we will provide all the data. We will provide all environmental data and epigenetic data.
