# Supplementary material for: Epigenetic architecture of Pseudotaxus chienii: Revealing the synergistic effects of climate and soil variables
Source: Ecol Evol. 2023 Sep 10;13(9):e10511. doi: 10.1002/ece3.10511 (PMC10493196; doi:10.1002/ece3.10511)
Supplement: Supplementary file 1 — Appendix S1: [file ECE3-13-e10511-s001.docx]

Appendix

**Supplementary Material**

**FIGURE S1**

**
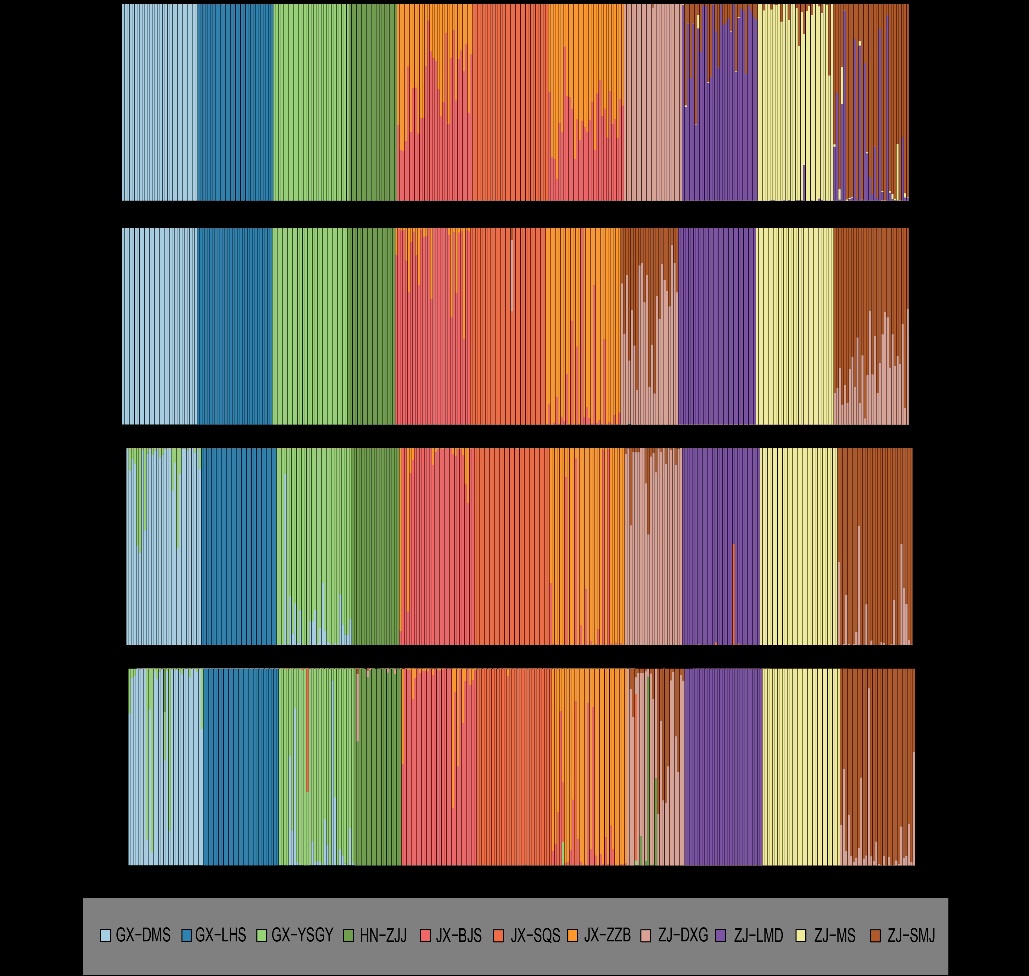
**

**FIGURE S2**

**
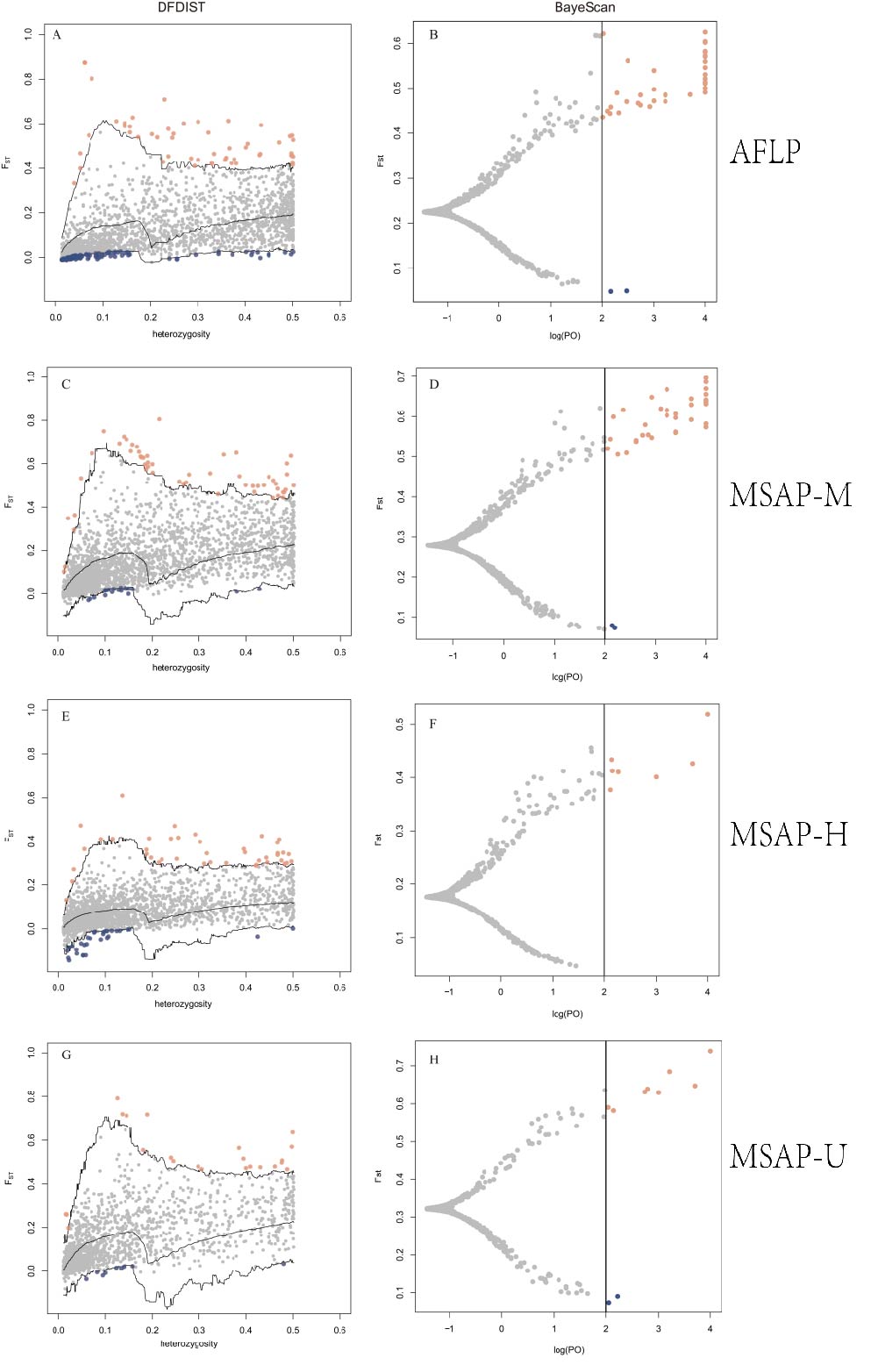
**

**FIGURE LEGENDS**

**FIGURE S1** Group assignment of individuals based on DAPC analysis. From top to bottom: AFLP, MSAP-M, MSAP-H, and MSAP-U.

**FIGURE S2** Outliers detected through DFDIST (left) and BAYESCAN (right). A and B: AFLP; C and D: MSAP-M; E and F: MSAP-H; G and H: MSAP-U. The orange dots represent the outliers under positive selection, while the blue dots stand for the outliers under negative selection.

**TABLE S1** Ten primer combinations used for amplified fragment length polymorphism (AFLP) in this study. Selective nucleotides for each primer were labeled in bold

| No. | *EcoR*I Primer | *Mse*I Primer |
| --- | --- | --- |
| 1 | 5′-GACTGCGTACCAATTC**AGC**-3′ | 5′-GATGAGTCCTGAGTAA**CAA**-3′ |
| 2 | 5′-GACTGCGTACCAATTC**AGC**-3′ | 5′-GATGAGTCCTGAGTAA**CAG**-3′ |
| 3 | 5′-GACTGCGTACCAATTC**AGC**-3′ | 5′-GATGAGTCCTGAGTAA**CTG**-3′ |
| 4 | 5′-GACTGCGTACCAATTC**AGG**-3′ | 5′-GATGAGTCCTGAGTAA**CAG**-3′ |
| 5 | 5′-GACTGCGTACCAATTC**AGG**-3′ | 5′-GATGAGTCCTGAGTAA**CAC**-3′ |
| 6 | 5′-GACTGCGTACCAATTC**AGG**-3′ | 5′-GATGAGTCCTGAGTAA**CTG**-3′ |
| 7 | 5′-GACTGCGTACCAATTC**AAC**-3′ | 5′-GATGAGTCCTGAGTAA**CAG**-3′ |
| 8 | 5′-GACTGCGTACCAATTC**AAC**-3′ | 5′-GATGAGTCCTGAGTAA**CTC**-3′ |
| 9 | 5′-GACTGCGTACCAATTC**AAG**-3′ | 5′-GATGAGTCCTGAGTAA**CAG**-3′ |
| 10 | 5′-GACTGCGTACCAATTC**ACG**-3′ | 5′-GATGAGTCCTGAGTAA**CAG**-3′ |

**TABLE S2** Information of adaptors and primers used in MSAP

|  | *Bgl* II（5-FAM） | *Tfi* I/*Pfe* I | |
| --- | --- | --- | --- |
| adaptors | 5′-CTCGTAGACTGCGTACC-3′  3′-CATCTGACGCATGGCTAG-5′ | | 5′-GACGATGAGTCTAGAA-3′  3′-CTACTCAGATCTTTWA-5′ |
| Primers of preamplification | 5′-GACTGCGTACCGATCTA-3′ | 5′-GATGAGTCTAGAAAWTCC-3′ | |
| Primers of selective amplification | 1：5′-GACTGCGTACCGATCTAGT-3′  1：5′-GACTGCGTACCGATCTAGT-3′  2：5′-GACTGCGTACCAATTCAGA-3′  2：5′-GACTGCGTACCAATTCAGA-3′  2：5′-GACTGCGTACCAATTCAGA-3′  2：5′-GACTGCGTACCAATTCAGA-3′  2：5′-GACTGCGTACCAATTCAGA-3′  4：5′-GACTGCGTACCGATCTATG-3′  5：5′-GACTGCGTACCGATCTAAC-3′  6：5′-GACTGCGTACCGATCTACA-3′  6：5′-GACTGCGTACCGATCTACA-3′  7：5′-GACTGCGTACCAATTCAAC-3′  8：5′-GACTGCGTACCAATTCAGC-3′  10：5′-GACTGCGTACCAATTCACA-3′  11：5′-GACTGCGTACCAATTCACG-3′  13：5′-GACTGCGTACCAATTCACT-3′  14：5′-GACTGCGTACCAATTCAAG-3′  15：5′-GACTGCGTACCAATTCAAT-3′  3：5′-GACTGCGTACCAATTCATT-3′  3：5′-GACTGCGTACCAATTCATT-3′  3：5′-GACTGCGTACCAATTCATT-3′ | 1：5′-GATGAGTCTAGAAAWTCCAT-3′  3：5′-GATGAGTCTAGAAAWTCCCT-3′  1：5′-GATGAGTCTAGAAAWTCCAT-3′  2：5′-GATGAGTCTAGAAAWTCCTT-3′  3：5′-GATGAGTCTAGAAAWTCCCT-3′  5：5′-GATGAGTCTAGAAAWTCCTG-3′  7：5′-GATGAGTCTAGAAAWTCCCA-3′  6：5′-GATGAGTCTAGAAAWTCCAG-3′  8：5′-GATGAGTCTAGAAAWTCCAT-3′  7：5′-GATGAGTCTAGAAAWTCCCA-3′  8：5′-GATGAGTCTAGAAAWTCCAT-3′  9：5′-GATGAGTCTAGAAAWTCCAA-3′  10：5′-GATGAGTCTAGAAAWTCCCC-3′  12：5′-GATGAGTCTAGAAAWTCCAC-3′  13：5′-GATGAGTCTAGAAAWTCCGC-3′  15：5′-GATGAGTCTAGAAAWTCCTG-3′  10：5′-GATGAGTCTAGAAAWTCCCC-3′  10：5′-GATGAGTCTAGAAAWTCCCC-3′  4：5′-GATGAGTCTAGAAAWTCCAC-3′  5：5′-GATGAGTCTAGAAAWTCCTG-3′  6：5′-GATGAGTCTAGAAAWTCCAG-3′ | |

**Table S3** Thirty-nine environmental variables and their abbreviations in the present study; the number of variables of each class is present in the parenthesis

| Classification | No. | Abbreviation | Interpretation |
| --- | --- | --- | --- |
| Climate Variables (19) | | | |
| Temperature (11) |  | Bio1 | Annual Mean Temperature |
|  |  | Bio2 | Mean Diurnal Range |
|  |  | Bio3 | Isothermality |
|  |  | Bio4 | Temperature Seasonality |
|  |  | Bio5 | Max Temperature of Warmest Month |
|  |  | Bio6 | Min Temperature of Coldest Month |
|  |  | Bio7 | Temperature Annual Range |
|  |  | Bio8 | Mean Temperature of Wettest Quarter |
|  |  | Bio9 | Mean Temperature of Driest Quarter |
|  |  | Bio10 | Mean Temperature of Warmest Quarter |
|  |  | Bio11 | Mean Temperature of Coldest Quarter |
| Precipitation (8) |  | Bio12 | Annual Precipitation |
|  |  | Bio13 | Precipitation of Wettest Month |
|  |  | Bio14 | Precipitation of Driest Month |
|  |  | Bio15 | Precipitation Seasonality |
|  |  | Bio16 | Precipitation of Wettest Quarter |
|  |  | Bio17 | Precipitation of Driest Quarter |
|  |  | Bio18 | Precipitation of Warmest Quarter |
|  |  | Bio19 | Precipitation of Coldest Quarter |
| Soil Variables (20) | | | |
| Physicochemical property (5) |  | Fresh Water | Water Content of Fresh Soil |
|  |  | Air-dried Water | Water Content of Air-dried Soil |
|  |  | pH | pH Value of Soil Suspension |
|  |  | EC | Electrical Conductivity of Soil Suspension |
|  |  | OM | Content of Organic Matter |
| Nonmetal (5) |  | N | Content of Nitrogen |
|  |  | C | Content of Carbon |
|  |  | P | Content of Phosphorus |
|  |  | S | Content of Sulfur |
|  |  | Si | Content of Silicon |
| Metal (10) |  | K | Content of Potassium |
|  |  | Ca | Content of Calcium |
|  |  | Na | Content of Sodium |
|  |  | Mg | Content of Magnesium |
|  |  | Al | Content of Aluminum |
|  |  | Fe | Content of Ferrum |
|  |  | Mn | Content of Manganese |
|  |  | Zn | Content of Zinc |
|  |  | Cu | Content of Cuprum |
|  |  | Pb | Content of Plumbum |

**Table S4** Climatic factors for 11 populations of *Pseudotaxus chienii*

| Pop | Temperature variables | | | | | | | | | | | Precipitation variables | | | | | | | |
| --- | --- | --- | --- | --- | --- | --- | --- | --- | --- | --- | --- | --- | --- | --- | --- | --- | --- | --- | --- |
|  | Bio1  °C | Bio2  °C | Bio3 | Bio4 | Bio5  °C | Bio6  °C | Bio7  °C | Bio8  °C | Bio9  °C | Bio10  °C | Bio11  °C | Bio12  mm | Bio13  mm | Bio14  mm | Bio15 | Bio16  mm | Bio17  mm | Bio18  mm | Bio19  mm |
| SMJ | 13.9 | 7.7 | 25.87 | 790.47 | 28.4 | -1.2 | 29.6 | 21.2 | 5.8 | 23.3 | 3.8 | 1,896 | 319 | 46 | 51.9 | 787 | 192 | 668 | 233 |
| LMD | 14.1 | 7.7 | 25.90 | 793.49 | 28.7 | -1.0 | 29.7 | 17.9 | 6.0 | 23.6 | 4.0 | 1,891 | 318 | 47 | 52.2 | 791 | 193 | 661 | 235 |
| MS | 15.8 | 8.4 | 28.46 | 766.12 | 30.1 | 0.7 | 29.4 | 19.4 | 8.0 | 24.9 | 6.0 | 1,909 | 331 | 45 | 54.7 | 809 | 183 | 678 | 232 |
| DXG | 11.8 | 7.2 | 26.88 | 696.24 | 24.6 | -2.0 | 26.6 | 18.3 | 4.7 | 20.1 | 2.9 | 2,119 | 337 | 47 | 51.8 | 842 | 193 | 792 | 233 |
| SQS | 13.3 | 7.3 | 25.32 | 779.97 | 27.1 | -1.6 | 28.7 | 17.0 | 5.2 | 22.5 | 3.3 | 2,081 | 345 | 53 | 54.7 | 911 | 203 | 730 | 255 |
| ZZB | 12.4 | 7.2 | 26.11 | 735.39 | 25.8 | -1.9 | 27.7 | 16.2 | 4.8 | 21.1 | 3.0 | 1,870 | 291 | 53 | 51.6 | 800 | 189 | 619 | 232 |
| BJS | 13.8 | 7.4 | 26.02 | 764.10 | 27.8 | -0.8 | 28.6 | 17.7 | 5.9 | 22.9 | 4.0 | 1,778 | 272 | 52 | 50.7 | 759 | 187 | 579 | 228 |
| Hnzjj | 13.8 | 7.6 | 25.73 | 801.28 | 28.6 | -0.9 | 29.5 | 21.4 | 3.6 | 23.5 | 3.6 | 1,490 | 230 | 35 | 56.3 | 642 | 119 | 601 | 119 |
| YS | 14.8 | 7.8 | 31.67 | 625.27 | 26.0 | 1.5 | 24.5 | 20.7 | 8.4 | 21.8 | 6.6 | 1,628 | 285 | 40 | 67.1 | 774 | 129 | 716 | 169 |
| LHS | 17.0 | 7.9 | 31.57 | 645.03 | 28.5 | 3.5 | 25.0 | 23.0 | 10.4 | 24.1 | 8.5 | 1,556 | 279 | 40 | 67 | 744 | 131 | 683 | 169 |
| DMS | 15.9 | 7.4 | 32.89 | 572.47 | 25.9 | 3.3 | 22.6 | 22.2 | 9.9 | 22.2 | 8.3 | 1,693 | 288 | 35 | 73.4 | 851 | 113 | 851 | 152 |
| Mean | 14.2 | 7.6 | 27.86 | 724.53 | 27.4 | 0.0 | 27.4 | 19.5 | 6.6 | 22.7 | 4.9 | 1,810 | 300 | 45 | 57.4 | 792 | 167 | 689 | 205 |

**TABLE S5** Eight ecology and topographic variables of 11 sampling sites

| Pop | EVI | FPAR | LAI | NDVI | PTC | ALT | Slope | Aspect |
| --- | --- | --- | --- | --- | --- | --- | --- | --- |
| DMS | 0.5133 | 0.9827 | 6.8000 | 0.8578 | 46.7500 | 1240 | 11.2852 | 35.5777 |
| LHS | 0.4809 | 0.9318 | 6.3273 | 0.8334 | 60.9167 | 1026 | 33.5373 | 75.2908 |
| YSGY | 0.5974 | 0.9645 | 6.6182 | 0.8907 | 75.8333 | 1050 | 22.1686 | 179.0720 |
| ZJJ | 0.5680 | 0.9564 | 6.0727 | 0.8393 | 53.1667 | 1055 | 39.2564 | 119.1530 |
| BJS | 0.5391 | 0.9909 | 6.7546 | 0.8967 | 70.2500 | 1340 | 12.1686 | 351.0360 |
| SQS | 0.5081 | 0.9609 | 6.0273 | 0.8601 | 65.7500 | 1500 | 17.2109 | 255.3580 |
| ZZB | 0.6367 | 0.9645 | 6.7909 | 0.8912 | 63.3333 | 1300 | 17.2109 | 255.3580 |
| DXG | 0.5178 | 0.9455 | 5.7364 | 0.8494 | 67.6667 | 1500 | 8.6687 | 266.0510 |
| LMD | 0.5387 | 0.9855 | 6.6455 | 0.8953 | 73.2500 | 1200 | 21.7330 | 223.0480 |
| MS | 0.5402 | 0.9573 | 6.0455 | 0.8474 | 57.4167 | 1120 | 21.3199 | 296.4270 |
| SMJ | 0.5867 | 0.9818 | 6.6091 | 0.8963 | 69.6667 | 830 | 4.2220 | 1.3972 |

**Table S6** Soil variables for 11 populations of *Pseudotaxus chienii*

| Pop | Fresh Water  % | Air-dried Water  % | pH | EC μS cm^-1^ | OM % | C % | N % | P  mg g^-1^ | S  mg g^-1^ | Si  mg g^-1^ | K mg g^-1^ | Ca  mg g^-1^ | Na mg g^-1^ | Mg  mg g^-1^ | Al mg g^-1^ | Fe  mg g^-1^ | Mn  mg g^-1^ | Zn mg g^-1^ | Cu  mg g^-1^ | Pb  mg g^-1^ |
| --- | --- | --- | --- | --- | --- | --- | --- | --- | --- | --- | --- | --- | --- | --- | --- | --- | --- | --- | --- | --- |
| SMJ | 53.69 | 10.9 | 3.95 | 117.90 | 26.48 | 29.585 | 0.651 | 0.698 | 1.376 | 72.578 | 3.939 | 3.741 | 13.021 | 1.606 | 16.979 | 20.724 | 0.313 | 0.358 | 0.010 | 0.077 |
| LMD | 52.72 | 11.36 | 3.63 | 173.40 | 32.76 | 35.743 | 0.692 | 0.553 | 1.427 | 146.724 | 3.380 | 6.304 | 13.125 | 1.884 | 16.302 | 12.494 | 0.837 | 0.276 | 0.008 | 0.074 |
| MS | 46.06 | 5.99 | 4.00 | 25.93 | 8.42 | 7.325 | 0.128 | 0.300 | 0.461 | 287.135 | 2.150 | 4.353 | 13.750 | 1.139 | 22.500 | 44.542 | 0.166 | 0.241 | 0.005 | 0.073 |
| DXG | 43.66 | 6.88 | 3.95 | 53.18 | 13.60 | 11.525 | 0.207 | 0.131 | 0.598 | 236.683 | 4.331 | 3.191 | 9.843 | 1.693 | 18.802 | 23.474 | 0.141 | 0.136 | 0.006 | 0.064 |
| SQS | 37.06 | 6.43 | 4.31 | 214.93 | 24.60 | 20.183 | 0.542 | 0.508 | 1.306 | 90.958 | 9.102 | 14.264 | 31.528 | 3.933 | 15.694 | 16.569 | 0.341 | 0.525 | 0.033 | 0.068 |
| ZZB | 55.60 | 8.55 | 3.23 | 290.07 | 28.97 | 26.130 | 0.587 | 0.743 | 1.583 | 311.451 | 5.601 | 25.887 | 43.403 | 9.052 | 20.417 | 13.382 | 0.369 | 0.888 | 0.022 | 0.093 |
| BJS | 47.06 | 8.21 | 3.52 | 231.77 | 27.73 | 17.910 | 0.474 | 0.696 | 1.166 | 98.125 | 5.874 | 17.881 | 17.500 | 4.901 | 12.847 | 11.292 | 0.234 | 0.739 | 0.024 | 0.039 |
| ZJJ | 54.34 | 9.17 | 3.12 | 181.80 | 18.50 | 17.773 | 0.466 | 0.831 | 1.198 | 281.097 | 6.733 | 21.617 | 20.486 | 4.241 | 19.694 | 24.660 | 0.388 | 1.137 | 0.024 | 0.087 |
| YS | 52.79 | 10.18 | 3.22 | 210.03 | 30.17 | 45.917 | 0.556 | 0.439 | 1.194 | 121.354 | 1.924 | 6.350 | 6.250 | 1.570 | 7.153 | 3.410 | 0.086 | 0.269 | 0.008 | 0.037 |
| LHS | 38.56 | 6.04 | 3.93 | 167.43 | 25.73 | 30.500 | 0.418 | 0.436 | 0.998 | 241.09 | 3.247 | 13.256 | 12.847 | 2.875 | 9.722 | 10.549 | 0.224 | 0.511 | 0.007 | 0.054 |
| DMS | 54.94 | 8.24 | 4.00 | 210.25 | 25.91 | 25.958 | 0.462 | 0.397 | 1.348 | 225.51 | 7.572 | 11.603 | 14.896 | 2.531 | 18.771 | 23.026 | 0.411 | 0.336 | 0.102 | 0.066 |
| Mean | 48.77 | 8.36 | 3.72 | 170.61 | 23.90 | 24.414 | 0.471 | 0.521 | 1.150 | 192.064 | 4.896 | 11.677 | 17.877 | 3.220 | 16.262 | 18.557 | 0.319 | 0.492 | 0.023 | 0.067 |

**TABLE S7** Proportions of different methylation status in each population

| Provenance | Population | Non-methylation（%） | Hemi-methylation（%） | Full-methylation（%） |
| --- | --- | --- | --- | --- |
| GX | DMS | 5.73% | 7.98% | 78.56% |
|  | LHS | 6.80% | 7.14% | 81.67% |
|  | YSGY | 6.30% | 9.70% | 75.87% |
|  | Mean | 6.28% | 8.28% | 78.70% |
| HN | ZJJ | 6.37% | 9.65% | 76.17% |
| JX | BJS | 7.97% | 10.47% | 71.72% |
|  | SQS | 7.24% | 12.02% | 71.42% |
|  | ZZB | 6.84% | 10.40% | 74.24% |
|  | Mean | 7.35% | 10.96% | 72.46% |
| ZJ | DXG | 5.94% | 12.75% | 75.30% |
|  | LMD | 8.84% | 11.37% | 70.77% |
|  | MS | 8.36% | 8.40% | 71.25% |
|  | SMJ | 6.68% | 8.86% | 79.23% |
|  | Mean | 7.57% | 10.19% | 74.01% |
| Total | | 7.06% | 9.83% | 75.04% |

**TABLE S8** Genetic and epigenetic parameters of different populations

| Datasets | Provenance | Pop | *Na* | *Ne* | *He* | Ia | rD |
| --- | --- | --- | --- | --- | --- | --- | --- |
| AFLP | GX | DMS | 1.558 | 1.320 | 0.196 | 15.655 | 0.0105 |
|  |  | LHS | 1.345 | 1.276 | 0.168 | 5.817 | 0.0047 |
|  |  | YSGY | 1.320 | 1.262 | 0.160 | 7.748 | 0.0063 |
|  | HN | ZJJ | 1.086 | 1.234 | 0.142 | 10.307 | 0.0102 |
|  | JX | BJS | 1.505 | 1.296 | 0.182 | 14.656 | 0.0104 |
|  |  | SQS | 1.514 | 1.307 | 0.188 | 11.215 | 0.0078 |
|  |  | ZZB | 1.343 | 1.266 | 0.163 | 10.417 | 0.0083 |
|  | ZJ | DXG | 1.344 | 1.280 | 0.170 | 9.938 | 0.0078 |
|  |  | LMD | 1.562 | 1.328 | 0.200 | 8.662 | 0.0059 |
|  |  | MS | 1.448 | 1.300 | 0.182 | 5.873 | 0.0043 |
|  |  | SMJ | 1.550 | 1.314 | 0.194 | 17.862 | 0.0120 |
|  |  | Average | 1.416 | 1.289 | 0.177 | 10.741 | 0.0080 |
| MSAP-M | GX | DMS | 1.355 | 1.246 | 0.144 | 8.480 | 0.0081 |
|  |  | LHS | 1.377 | 1.252 | 0.147 | 9.070 | 0.0108 |
|  |  | YSGY | 1.476 | 1.331 | 0.192 | 18.190 | 0.0152 |
|  | HN | ZJJ | 1.522 | 1.363 | 0.208 | 16.820 | 0.0141 |
|  | JX | BJS | 1.454 | 1.298 | 0.174 | 7.000 | 0.0064 |
|  |  | SQS | 1.588 | 1.380 | 0.222 | 10.810 | 0.0089 |
|  |  | ZZB | 1.560 | 1.389 | 0.224 | 19.280 | 0.0143 |
|  | ZJ | DXG | 1.479 | 1.313 | 0.182 | 8.130 | 0.0089 |
|  |  | LMD | 1.505 | 1.339 | 0.198 | 9.660 | 0.0078 |
|  |  | MS | 1.586 | 1.379 | 0.221 | 12.220 | 0.0078 |
|  |  | SMJ | 1.527 | 1.367 | 0.212 | 12.050 | 0.0101 |
|  |  | Average | 1.493 | 1.332 | 0.193 | 11.974 | 0.0102 |
| MSAP-H | GX | DMS | 0.809 | 1.116 | 0.076 | 4.731 | 0.0092 |
|  |  | LHS | 0.845 | 1.108 | 0.074 | 3.820 | 0.0067 |
|  |  | YSGY | 1.053 | 1.142 | 0.096 | 5.387 | 0.0077 |
|  | HN | ZJJ | 1.052 | 1.148 | 0.101 | 4.450 | 0.0064 |
|  | JX | BJS | 0.993 | 1.156 | 0.102 | 4.746 | 0.0074 |
|  |  | SQS | 1.214 | 1.182 | 0.120 | 4.985 | 0.0062 |
|  |  | ZZB | 1.176 | 1.156 | 0.107 | 6.860 | 0.0083 |
|  | ZJ | DXG | 1.067 | 1.186 | 0.119 | 6.972 | 0.0123 |
|  |  | LMD | 1.131 | 1.169 | 0.112 | 6.570 | 0.0080 |
|  |  | MS | 1.158 | 1.130 | 0.092 | 5.300 | 0.0054 |
|  |  | SMJ | 1.114 | 1.133 | 0.094 | 4.191 | 0.0053 |
|  |  | Average | 1.056 | 1.148 | 0.099 | 5.274 | 0.0075 |
| MSAP-U | GX | DMS | 0.555 | 1.096 | 0.060 | 2.730 | 0.0069 |
|  |  | LHS | 0.681 | 1.114 | 0.071 | 4.310 | 0.0099 |
|  |  | YSGY | 0.775 | 1.122 | 0.078 | 8.610 | 0.0165 |
|  | HN | ZJJ | 0.721 | 1.132 | 0.084 | 7.670 | 0.0153 |
|  | JX | BJS | 0.805 | 1.149 | 0.092 | 5.330 | 0.0092 |
|  |  | SQS | 0.898 | 1.149 | 0.094 | 5.090 | 0.0081 |
|  |  | ZZB | 0.875 | 1.132 | 0.085 | 6.520 | 0.0111 |
|  | ZJ | DXG | 0.680 | 1.121 | 0.076 | 9.270 | 0.0223 |
|  |  | LMD | 0.882 | 1.156 | 0.098 | 5.010 | 0.0082 |
|  |  | MS | 1.031 | 1.181 | 0.113 | 10.970 | 0.0135 |
|  |  | SMJ | 0.838 | 1.125 | 0.081 | 3.800 | 0.0070 |
|  |  | Average | 0.795 | 1.134 | 0.085 | 6.301 | 0.0116 |

Note：*Na*, Number of different alleles; *Ne*, Number of effective alleles; *He*, Expected heterozygosity; Ia, Index of association; rD, Modified index of association.

**TABLE S9** Pairwise *F_ST_* values between populations

|  | DMS | LHS | YSGY | ZJJ | BJS | SQS | ZZB | DXG | LMD | MS | SMJ |
| --- | --- | --- | --- | --- | --- | --- | --- | --- | --- | --- | --- |
| AFLP |  |  |  |  |  |  |  |  |  |  |  |
| DMS | 0.000 |  |  |  |  |  |  |  |  |  |  |
| LHS | 0.2236 | 0.0000 |  |  |  |  |  |  |  |  |  |
| YSGY | 0.2297 | 0.1579 | 0.000 |  |  |  |  |  |  |  |  |
| ZJJ | 0.3179 | 0.3600 | 0.3423 | 0.0000 |  |  |  |  |  |  |  |
| BJS | 0.2030 | 0.2644 | 0.2592 | 0.3071 | 0.0000 |  |  |  |  |  |  |
| SQS | 0.2172 | 0.3098 | 0.2967 | 0.3277 | 0.2101 | 0.0000 |  |  |  |  |  |
| ZZB | 0.2405 | 0.2753 | 0.2454 | 0.2982 | 0.0526 | 0.2441 | 0.0000 |  |  |  |  |
| DXG | 0.2517 | 0.3475 | 0.3310 | 0.3550 | 0.2546 | 0.2046 | 0.2728 | 0.0000 |  |  |  |
| LMD | 0.2187 | 0.2896 | 0.2861 | 0.3324 | 0.2101 | 0.1783 | 0.2440 | 0.1277 | 0.0000 |  |  |
| MS | 0.2386 | 0.2987 | 0.2926 | 0.3289 | 0.2281 | 0.1980 | 0.2434 | 0.1315 | 0.0895 | 0.0000 |  |
| SMJ | 0.1908 | 0.2718 | 0.2476 | 0.2868 | 0.1864 | 0.1317 | 0.2060 | 0.1161 | 0.0632 | 0.1209 | 0.0000 |
|  |  |  |  |  |  |  |  |  |  |  |  |
| **MSAP-M** |  |  |  |  |  |  |  |  |  |  |  |
| DMS | 0.0000 |  |  |  |  |  |  |  |  |  |  |
| LHS | 0.3811 | 0.0000 |  |  |  |  |  |  |  |  |  |
| YSGY | 0.1760 | 0.2827 | 0.000 |  |  |  |  |  |  |  |  |
| ZJJ | 0.2993 | 0.3639 | 0.2364 | 0.0000 |  |  |  |  |  |  |  |
| BJS | 0.3422 | 0.4220 | 0.2720 | 0.2663 | 0.0000 |  |  |  |  |  |  |
| SQS | 0.2899 | 0.3757 | 0.2379 | 0.2554 | 0.2547 | 0.0000 |  |  |  |  |  |
| ZZB | 0.2544 | 0.3434 | 0.2035 | 0.2287 | 0.0955 | 0.1273 | 0.0000 |  |  |  |  |
| DXG | 0.3527 | 0.4299 | 0.2812 | 0.2356 | 0.3089 | 0.2482 | 0.2556 | 0.0000 |  |  |  |
| LMD | 0.3215 | 0.3804 | 0.2099 | 0.3018 | 0.2875 | 0.2151 | 0.2346 | 0.2746 | 0.0000 |  |  |
| MS | 0.2969 | 0.3550 | 0.2200 | 0.2609 | 0.2702 | 0.2096 | 0.2187 | 0.2435 | 0.1626 | 0.0000 |  |
| SMJ | 0.2842 | 0.2117 | 0.2211 | 0.2357 | 0.2968 | 0.2445 | 0.2313 | 0.1957 | 0.2194 | 0.2056 | 0.0000 |
|  |  |  |  |  |  |  |  |  |  |  |  |
| **MSAP-H** |  |  |  |  |  |  |  |  |  |  |  |
| DMS | 0.0000 |  |  |  |  |  |  |  |  |  |  |
| LHS | 0.2827 | 0.0000 |  |  |  |  |  |  |  |  |  |
| YSGY | 0.1375 | 0.2057 | 0.000 |  |  |  |  |  |  |  |  |
| ZJJ | 0.2350 | 0.2632 | 0.1800 | 0.0000 |  |  |  |  |  |  |  |
| BJS | 0.2654 | 0.3157 | 0.2035 | 0.1874 | 0.0000 |  |  |  |  |  |  |
| SQS | 0.2322 | 0.2559 | 0.1858 | 0.1838 | 0.1992 | 0.0000 |  |  |  |  |  |
| ZZB | 0.1998 | 0.2539 | 0.1526 | 0.1559 | 0.0801 | 0.0946 | 0.0000 |  |  |  |  |
| DXG | 0.2928 | 0.3572 | 0.2247 | 0.1810 | 0.2463 | 0.2061 | 0.2035 | 0.0000 |  |  |  |
| LMD | 0.2473 | 0.2742 | 0.1433 | 0.2091 | 0.2064 | 0.1650 | 0.1642 | 0.2074 | 0.0000 |  |  |
| MS | 0.2074 | 0.2264 | 0.1450 | 0.1731 | 0.2023 | 0.1692 | 0.1613 | 0.2051 | 0.1150 | 0.0000 |  |
| SMJ | 0.2126 | 0.1372 | 0.1548 | 0.1531 | 0.2140 | 0.1940 | 0.1659 | 0.1591 | 0.1601 | 0.1337 | 0.0000 |
|  |  |  |  |  |  |  |  |  |  |  |  |
| **MSAP-U** |  |  |  |  |  |  |  |  |  |  |  |
| DMS | 0.0000 |  |  |  |  |  |  |  |  |  |  |
| LHS | 0.3264 | 0.0000 |  |  |  |  |  |  |  |  |  |
| YSGY | 0.1414 | 0.2263 | 0.000 |  |  |  |  |  |  |  |  |
| ZJJ | 0.2961 | 0.3336 | 0.2382 | 0.0000 |  |  |  |  |  |  |  |
| BJS | 0.2917 | 0.3923 | 0.2550 | 0.2330 | 0.0000 |  |  |  |  |  |  |
| SQS | 0.2257 | 0.2756 | 0.1875 | 0.2516 | 0.2450 | 0.0000 |  |  |  |  |  |
| ZZB | 0.2184 | 0.2613 | 0.1638 | 0.1906 | 0.0916 | 0.1234 | 0.0000 |  |  |  |  |
| DXG | 0.3143 | 0.3496 | 0.2456 | 0.2167 | 0.2833 | 0.2424 | 0.2100 | 0.0000 |  |  |  |
| LMD | 0.3112 | 0.3124 | 0.2096 | 0.2982 | 0.3093 | 0.2268 | 0.2185 | 0.2792 | 0.0000 |  |  |
| MS | 0.2942 | 0.3268 | 0.2484 | 0.2974 | 0.3192 | 0.2390 | 0.2435 | 0.2824 | 0.2102 | 0.0000 |  |
| SMJ | 0.2685 | 0.1772 | 0.2018 | 0.2388 | 0.2961 | 0.1800 | 0.1688 | 0.1704 | 0.1720 | 0.2325 | 0.0000 |

**Table S10** Correlation analysis of geography, environment and their joint effect based on multiple matrix regression with randomization (MMRR)

|  | Environment and Geography | | Geography | | Environment | |
| --- | --- | --- | --- | --- | --- | --- |
|  | *F* | *p* | correlation coefficient | *p* | correlation coefficient | *p* |
| AFLP | 25.172 | 0.001 | 9.91×10^-5^ | 0.001 | 0.423 | 0.002 |
| MSAP-M | 0.869 | 0.514 | 7.42×10^-6^ | 0.191 | -4.31×10^-5^ | 0.994 |
| MSAP-H | 0.777 | 0.542 | 8.65×10^-6^ | 0.238 | 0.4 | 0.509 |
| MSAP-U | 0.71 | 0.568 | 6.28×10^-6^ | 0.240 | 1.276 | 0.24 |

**TABLE S11** Relationship between environmental variables and outliers based on generalized linear mixed model (GLMM)

| Loci | Bio10 | Bio11 | Bio13 | Bio14 | Bio16 | PTC | LAI | fPAR | EVI | ALT | Slope | Aspect | K | Na | Fe | Mn | Zn | Cu | Pb |
| --- | --- | --- | --- | --- | --- | --- | --- | --- | --- | --- | --- | --- | --- | --- | --- | --- | --- | --- | --- |
| **AFLP** |  |  |  |  |  |  |  |  |  |  |  |  |  |  |  |  |  |  |  |
| 223 |  |  |  | *** | * | *** |  | ** | * | *** |  |  | ** |  | ** | *** |  | *** |  |
| 249 | ** |  | *** | *** | *** | *** | * |  | *** | *** | ** |  | *** | ** | *** | *** | *** | *** | *** |
| 301 | *** |  | *** | *** | ** | *** | *** | *** | *** |  | *** |  |  |  | * | ** | *** | *** |  |
| 318 |  |  |  |  |  |  |  |  |  |  |  |  |  |  |  |  |  |  |  |
| 425 |  |  |  |  |  |  |  |  |  |  |  |  |  |  |  |  |  |  |  |
| 436 |  |  | *** |  | *** | * | ** |  |  | * | *** |  | *** |  | *** |  | *** | ** |  |
| 554 |  |  | *** |  | ** | *** | *** | ** |  | * |  |  | * | *** | *** |  |  |  | *** |
| 583 | *** | *** | *** |  | * | *** | * |  | ** |  |  | *** |  | ** | ** | *** | *** |  | ** |
| 842 | *** | ** | *** | *** | *** | * | *** | *** | ** | *** | *** |  | *** |  | *** | ** | *** | *** |  |
| 894 |  | *** |  | *** |  | *** |  |  | ** |  |  | *** | *** | *** | *** | *** | *** | *** | *** |
| 910 | ** | * | *** | ** | *** | *** | *** |  | *** | *** |  | * | *** | * | *** | *** | *** | *** | ** |
| 948 | *** | *** | *** | *** | *** | *** |  |  | *** | *** | * |  | *** |  | *** | *** | *** |  |  |
| 1063 |  |  | * | *** | *** | *** | * | *** | * | *** | *** |  | *** |  | *** | *** | ** |  |  |
| 1081 | * | * |  |  |  |  |  |  |  |  |  |  |  |  |  |  | * |  |  |
| 1116 | ** |  | *** |  | *** | *** | *** |  | *** | ** |  |  | *** |  | *** |  | *** | ** |  |
| 1121 |  |  | * | *** | *** | ** | ** | *** |  |  | *** | * | *** |  | *** |  | * | *** | * |
| 1339 |  |  |  |  |  |  |  |  |  |  |  |  |  |  |  |  |  |  |  |
| 1341 |  |  |  |  |  |  |  |  |  |  |  |  |  |  |  |  |  |  |  |
| 1682 |  |  | *** | *** | *** | *** | ** | * | *** | *** | *** |  | *** |  | *** | *** | *** | *** | ** |
| 1710 |  | * | *** | *** | *** | *** | *** | * | *** | *** | *** | *** | *** |  | *** | *** | *** | *** |  |
| 1711 |  | * | *** | *** | *** | *** | *** | * | *** | *** | *** | *** | *** |  | *** | *** | *** | *** |  |
| 1742 |  |  | *** | * | *** | *** | *** |  | *** | *** | *** |  | *** |  | ** | *** | *** | *** |  |
| 1893 | * |  | *** | *** | *** | *** |  | *** | *** | *** | *** | ** | *** |  | *** | *** | *** | *** | * |
| 1899 | * |  |  |  | *** | *** |  | *** |  | * | *** | * | *** |  |  | ** |  | *** | ** |
| 1996 | ** | *** | * | * | ** | * |  |  |  |  | * |  | *** |  | ** | * |  |  |  |
| **MSAP-M** |  |  |  |  |  |  |  |  |  |  |  |  |  |  |  |  |  |  |  |
| B59 |  | * |  |  |  |  | ** |  |  |  |  |  |  |  | ** | . |  |  | * |
| D205 |  |  |  | * |  | *** | * | * |  |  | ** | ** | *** | *** | *** | ** | ** | *** | * |
| D247 |  |  |  |  |  |  |  |  |  |  |  |  |  |  |  |  |  |  |  |
| I88 |  |  |  | ** |  | * |  |  |  |  |  |  |  | * | *** | *** |  |  |  |
| I143 |  |  |  |  |  |  |  |  |  |  |  |  |  |  |  |  |  |  |  |
| I147 |  |  |  |  |  |  |  |  |  |  |  |  | . |  |  |  |  |  |  |
| I205 |  |  |  | * |  |  |  |  |  |  | ** |  |  | *** |  | ** |  |  | . |
| J83 |  |  |  |  |  |  |  |  | * |  |  |  | ** |  | * |  |  | * |  |
| K83 |  |  |  |  |  | *** | ** | * |  |  |  |  | * | * | *** | *** | * | * |  |
| K86 |  |  |  |  |  |  |  |  |  |  |  |  |  |  |  |  |  |  |  |
| K148 |  | * |  | ** |  | ** | ** |  |  |  |  | * | * |  |  |  |  | ** |  |
| M109 | *** |  | *** | * | *** |  | *** |  |  |  |  | * |  |  | *** |  | *** |  |  |
| M162 | *** | *** | *** | ** |  |  | ** | ** | ** |  | *** | *** | *** |  |  |  | *** | *** |  |
| N217 |  |  |  |  |  |  | ** | ** |  |  | * | ** |  |  |  |  |  | * |  |
| N323 |  | * |  | *** |  | *** |  |  |  |  |  |  |  | * | *** |  |  | *** | *** |
| N425 | * |  | * |  |  | * |  |  |  |  |  | * |  |  | ** |  |  |  |  |
| N426 | *** | ** |  |  |  |  | ** | *** | *** |  | ** |  |  |  |  |  |  | * |  |
| N485 | ** |  | ** |  | ** | * | ** |  | * |  |  |  |  |  | *** |  |  |  | . |
| O101 |  |  |  |  |  |  | * | ** |  |  | * |  |  |  |  |  |  | . |  |
| O222 |  |  |  |  |  |  | ** | ** |  |  |  |  | ** | ** |  | ** |  | *** |  |
| Q323 | * | * |  |  |  |  | ** | ** |  |  |  | * | * |  |  | ** |  | *** |  |
| R267 |  |  |  |  |  |  |  |  | * |  |  |  |  | ** |  |  | * |  | * |
| T199 | * |  | ** |  | * | * |  |  |  |  |  |  |  |  | ** |  | * |  |  |
| T200 |  |  | ** |  | ** | * | * |  |  |  |  | * | . | ** | *** |  |  |  | *** |
| T257 | * | ** |  |  |  | * |  |  | *** |  |  | * |  | * | * |  | . |  | * |
| U151 |  |  |  | * |  |  |  |  |  |  |  |  |  |  |  | * |  |  |  |
| **MSAP-H** |  |  |  |  |  |  |  |  |  |  |  |  |  |  |  |  |  |  |  |
| K91 |  |  |  |  |  |  |  |  |  |  |  |  |  |  |  | * |  | * |  |
| K111 |  | ** |  | *** | ** | *** |  | *** |  | *** | ** | *** | ** |  | *** |  | * | *** |  |
| K201 | *** |  | *** | *** | *** | *** |  | *** | *** | *** | *** |  | *** |  | ** | *** | *** | *** | ** |
| L150 |  |  | * | ** | * | ** |  |  |  |  |  |  |  |  | * |  |  |  |  |
| L175 |  |  |  |  |  |  |  |  | * |  |  |  |  |  |  |  |  |  |  |
| N55 | ** | ** | *** | *** | *** |  |  |  | ** | *** |  |  | *** | * |  | *** | *** | ** | * |
| N117 |  |  |  |  |  |  |  |  |  |  |  |  |  |  |  | * |  | * |  |
| **MSAP-U** |  |  |  |  |  |  |  |  |  |  |  |  |  |  |  |  |  |  |  |
| A95 |  |  |  | *** | *** |  | *** | *** | *** |  | ** |  | * |  |  | ** | ** | *** | * |
| D247 |  |  |  |  |  |  |  |  |  |  |  |  |  |  |  |  |  |  |  |
| J83 |  |  |  |  |  |  |  |  | * |  |  |  | ** |  | * |  |  | ** |  |
| M74 | *** |  |  |  | * |  |  | ** |  | * | *** |  | . |  |  |  |  | * |  |
| M162 | *** | *** | *** | ** |  |  | ** | ** | ** |  | *** | *** | *** |  |  |  | *** | *** |  |
| Q117 | * |  |  |  |  |  |  |  |  |  | * | * |  |  |  | ** |  | * |  |

*, ** and *** represent the significant code *p*<0.01， *p*<0.001 and *p*=0 respectively.

| Provenance | POP | PCNM1 | PCNM2 | PCNM3 | PCNM4 | PCNM5 | PCNM6 | PCNM7 |
| --- | --- | --- | --- | --- | --- | --- | --- | --- |
| GX | DMS | -0.2740 | 0.5334 | 0.0073 | -0.3898 | 0.5264 | 0.1592 | 0.0211 |
|  | LHS | -0.3401 | 0.3436 | -0.0572 | 0.6273 | 0.0066 | 0.0032 | 0.0006 |
|  | YSGY | -0.3815 | 0.1408 | 0.2695 | -0.1911 | -0.5322 | -0.1633 | -0.0220 |
| HN | ZJJ | -0.1363 | -0.2872 | -0.7895 | -0.3156 | -0.0042 | -0.0025 | -0.0005 |
| JX | BJS | -0.1847 | -0.4424 | 0.4792 | -0.3401 | -0.0045 | -0.0003 | 0.0000 |
|  | SQS | 0.2360 | -0.2883 | 0.2468 | 0.1797 | 0.5323 | 0.1609 | 0.0215 |
|  | ZZB | -0.2913 | -0.4160 | -0.0970 | 0.4128 | 0.0061 | 0.0029 | 0.0005 |
| ZJ | DXG | 0.3427 | 0.1043 | -0.0152 | 0.0029 | -0.3405 | 0.5669 | 0.5762 |
|  | LMD | 0.3431 | 0.1038 | -0.0145 | 0.0049 | 0.0060 | -0.5075 | 0.0787 |
|  | MS | 0.3430 | 0.1041 | -0.0148 | 0.0040 | -0.2004 | 0.2897 | -0.8027 |
|  | SMJ | 0.3431 | 0.1038 | -0.0145 | 0.0049 | 0.0045 | -0.5091 | 0.1268 |

**TABLE S12** Spatial predictors extracted from distance-based Moran’s eigenvector maps of 11 *Pseudotaxus chienii* populations
